# Identification of Serum MicroRNA Signatures for Diagnosis of Mild Traumatic Brain Injury in a Closed Head Injury Model

**DOI:** 10.1371/journal.pone.0112019

**Published:** 2014-11-07

**Authors:** Anuj Sharma, Raghavendar Chandran, Erin S. Barry, Manish Bhomia, Mary Anne Hutchison, Nagaraja S. Balakathiresan, Neil E. Grunberg, Radha K. Maheshwari

**Affiliations:** 1 Department of Pathology, Uniformed Services University of the Health Sciences, Bethesda, Maryland, United States of America; 2 Biological Sciences Group, Birla Institute of Technology and Science, Pilani, Rajasthan, India; 3 Department of Medical and Clinical Psychology, Uniformed Services University of the Health Sciences, Bethesda, Maryland, United States of America; East Carolina University, United States of America

## Abstract

Wars in Iraq and Afghanistan have highlighted the problems of diagnosis and treatment of mild traumatic brain injury (mTBI). MTBI is a heterogeneous injury that may lead to the development of neurological and behavioral disorders. In the absence of specific diagnostic markers, mTBI is often unnoticed or misdiagnosed. In this study, mice were induced with increasing levels of mTBI and microRNA (miRNA) changes in the serum were determined. MTBI was induced by varying weight and fall height of the impactor rod resulting in four different severity grades of the mTBI. Injuries were characterized as mild by assessing with the neurobehavioral severity scale-revised (NSS-R) at day 1 post injury. Open field locomotion and acoustic startle response showed behavioral and sensory motor deficits in 3 of the 4 injury groups at day 1 post injury. All of the animals recovered after day 1 with no significant neurobehavioral alteration by day 30 post injury. Serum microRNA (miRNA) profiles clearly differentiated injured from uninjured animals. Overall, the number of miRNAs that were significantly modulated in injured animals over the sham controls increased with the severity of the injury. Thirteen miRNAs were found to identify mTBI regardless of its severity within the mild spectrum of injury. Bioinformatics analyses revealed that the more severe brain injuries were associated with a greater number of miRNAs involved in brain related functions. The evaluation of serum miRNA may help to identify the severity of brain injury and the risk of developing adverse effects after TBI.

## Introduction

Traumatic brain injury (TBI) is one of the signature injuries in the conflicts with Iraq and Afghanistan [1]. Thirty percent of combat troops of Operation Iraqi Freedom (OIF) and Operation Enduring Freedom (OEF) have been diagnosed with mild, moderate, or severe TBI. More TBI cases occur out of the combat zone due to vehicle crashes, falls, sports, and recreational activities, which account for 84% of the total military TBI cases (www.dvbic.org). The Glasgow Coma Scale (GCS) is a standard measure to ascertain the initial severity and prognosis of TBI [2]. Mild TBI (mTBI), which is the most common form of military and civilian TBI, is characterized by loss of consciousness for <30 min and post traumatic amnesia for <24 hr with a GCS of 13–15, and accounts for 77% of the total TBI cases [3]. Diagnosis of mTBI is difficult as the injury may go unnoticed due to the lack of any immediate symptoms and confirmed pathology. Computed tomography (CT) and magnetic resonance imaging (MRI) have limited ability to detect mild brain tissue damage [4]–[6]. Serum levels of brain specific/enriched proteins such as S-100β, glial fibrillary acidic protein (GFAP) and its breakdown products, and ubiquitin carboxyl-terminal esterase L1 (UCH-L1) have been proposed as diagnostic markers of severe TBI, but their utility in the diagnosis of mild to moderate TBI is still unknown [7]–[12]. The majority of mild brain injuries recover spontaneously. However, 10–20% of mTBI patients continue to suffer from post concussive syndrome [3], [13]–[15]. Therefore, it is important to establish diagnostic marker(s) that can distinguish patients with a head injury regardless of the severity of the injury as well as distinguish individuals based on severity of mTBI.

MicroRNAs (miRNA), which are small (19–28 nt) non-coding endogenous RNA, have shown great promise as diagnostic markers of several diseases and disorders [16]–[18]. Unique changes in the expression of miRNAs in the brain samples after TBI have been reported [19]–[23]. Redell and group have also investigated the diagnostic potential of the miRNA in severe TBI [20]. To date, a comprehensive evaluation of miRNA as a diagnostic biomarker of mTBI, which also addresses the heterogeneous nature of mTBI, has not been described. The present study was designed to identify serum miRNAs as biomarkers of mild brain injury, which could identify the occurrence of mTBI independent of the severity of the injury within the mild spectrum of TBI.

In this study, a previously described free-fall weight-drop model of mTBI [25] with modifications was used to study the neurobehavioral deficits over a period of 30 days and miRNA modulation during the acute phase of injury. This model resembles the blunt head trauma resulting from a vehicle crash, combat, falls, and other recreational activities. Mice were subjected to an increasing grade of injury within the mild spectrum by varying the weight and height of the falling metal rod, with the most severe being a 3 cm fall height combined with a 333 g weight [25]. The neurobehavioral severity scale-revised (NSS-R) was measured at day 1 post injury, along with open field locomotion (OFL) activity and acoustic startle responses (ASR). NSS-R scores correlated and increased significantly with the grade of injury. OFL activity and startle responses were reduced in the injury groups, except the 2461g/2 cm injury. OFL and ASR activity returned to normal levels by day 14 post injury and remained constant through day 30 post injury measurements. To determine the miRNA changes in the serum during the acute phase of injury, miRNA arrays were performed on serum RNA isolated at 3 hr post injury. Thirteen miRNAs showed similar expression changes among injured mice compared to sham controls. Bioinformatics analyses using the Ingenuity Pathway Analysis (IPA) and DNA Intelligent Analysis (DIANA)-miRPath software showed that the number of significantly modulated serum miRNAs predicted to be involved in brain related functions increased with the severity of the injury.

## Material and Methods

### Animals and Injury

10–11 weeks old C57Bl/6J (Stock# 000664) male mice were obtained from Jackson Laboratories (Bar Harbor, ME). The animals were acclimatized to their new environment for a week and had continuous access to food and water. During the acclimatization period and throughout the experiment, the animals were housed on a reversed light cycle and were handled daily by the laboratory staff. The reverse light cycle was used because mice are nocturnal animals, so the behavioral measures were made during their active periods. Daily handling was done to habituate animals to laboratory staff and handling procedures, thereby reducing any stress associated with handling the animals during the experimental procedures.

#### Ethics Statement

All of the animal experiments were reviewed and approved by the Uniformed Services University of the Health Sciences (USUHS) Institutional Animal Care and Use Committee and were performed in accordance with the Guide for the Care and Use of Laboratory Animals (Committee on Care And Use of Laboratory Animals of The Institute of Laboratory Animal Resources, National Research Council, NIH Publication No. 86-23, revised 1996).

A weight drop injury apparatus (Figure S1A) was custom made by FJB Engineering (330-i North Stone Street Ave., Rockville, MD 20850) based on the description given in Flierl *et al.* (2009) [25]. The mice were anesthetized using a continuous flow of isoflurane/oxygen mix. The hair on the head was shaved and the animals were placed on the platform where continuous anesthesia was provided through a nose cone. The skin was sterilized using 10% povidone-iodine (Betadine) solution (Purdue Products L.P., Stamford, CT). A single cut was made along the mid line of the head to expose the skull. Injury coordinates were: left parietal lobe, 2.5 mm from sagittal suture, and 2.5 mm from lamboid suture (Figure S1B). Injury was induced by a free falling metal rod with a rubber tip of 1 mm diameter (Figure S1A). Rebound injury was minimized by immediately catching the rod after the primary impact. Animals were resuscitated by gently massaging the thorax and providing oxygen through the nose cone. Once continuous breathing resumed, the animals were again put on mild anesthesia using the nose cone. Animals without skull fractures were included in the experiment and the scalp skin was sutured back using Vetbond Tissue Adhesive 1469SB (3 M, St.Paul, MN, USA). The animals were then placed in a cage on a warming mat and were allowed to recuperate from the anesthesia. Injury day was considered as day 0. Sham controls were similarly handled and received only the scalp cut under anesthesia.

### Study Design

The experiment was designed to evaluate the neurobehavioral alteration in the mice following mTBI over a 30 day period and to evaluate the serum miRNA changes of mice at 3 hr post injury.

#### Behavior Study

Multiple smaller cohorts were combined to complete the final analyses. Four different injury groups: 246 g/2 cm (n = 32), 246 g/3 cm (n = 29), 333 g/2 cm (n = 20) and 333 g/3 cm (n = 6) were based on the weight of the impact rod and the height of the fall, respectively. These groups are referred to as injury severity 1 (IS1  = 246 g/2 cm), injury severity 2 (IS2  = 246 g/3 cm), injury severity 3 (IS3  = 333 g/2 cm), and injury severity 4 (IS4  = 333 g/3 cm) through rest of the manuscript. Sham controls (n = 42) were included in the experiment to determine the effect of handling and surgery (scalp cut and suture) on the animals' neurobehavioral responses. In the sham group, animals were handled similar to that of the injured groups except that no injury was made after the scalp cut. The skin was sutured back without making the impact trauma and the animals were allowed to recuperate in the cage similar to the injured animals. An additional control of naïve (n = 25) animals was included to determine the basal level of normal neurobehavioral responses. The naïve group of animals was kept in their housing room and not taken to the surgery room to avoid any stress related to being in the surgery room. Baseline measurements for NSS-R, OFL, and ASR were taken at day 3 (day −3) before the injury (day 0). NSS-R was also measured at day 1 post injury and ASR and OFL were measured at days 1, 14, and 30 post injury.

#### MicroRNA Study

A separate group of animals for all four injuries as described above in the behavior study, were used in the miRNA study (n = 6 each group). In addition, a sham group (n = 6) was included to account for miRNA changes that may occur due to the surgical process such as anesthesia, scalp cut, stress of handling the animal, and sutures. MiRNA expression of injured animals was compared with that of the sham to determine the modulation in miRNA expression due to brain injury.

### Neurobehavioral severity scale-revised (NSS-R)

A revised form of the neurological severity scale (NSS), initially developed for rats, was modified for mice and measured 3 days before the injury (day −3) and at day 1 post injury [26]–[27]. The NSS-R is a specific, continuous sequence of 10 behavioral tests and observations. This measure was originally designed to model a clinical neurological exam conducted in human neurology patients. This particular sensory-motor assessment scale was based on several previous reports and has been modified to increase standardization and sensitivity [27]–[32]. Ten tasks assess reflex suppression, general movement, and postural adjustments in response to a challenge. The NSS-R uses a three-point Likert scale, in which a normal, healthy response is assigned a “0,” a partial or compromised response is assigned a “1,” and the absence of a response is assigned a “2.” This three-point scale is clear and reliable and allows for greater discrimination based on sensory-motor responses than do previous scales that used two-point ratings of each response. The NSS-R has a scoring range of 0–20, with higher scores reflecting greater extent of injury. All personnel were blinded to the treatment groups for the NSS-R measurements, but not to the naïve group. Naïve animals did not exhibit a scalp cut and sutures, which were otherwise were present on all the sham and injured animals, and therefore, were easily identifiable. Animals were evaluated on all 10 tasks as listed in Table S1.

### Open Field Locomotion (OFL) test

Open field locomotor activity was measured to evaluate the unconditional behavior of the mouse in its environment. OFL test was performed at 3 days prior to injury (baseline; day −3) and days 1, 14, and 30 post injury. Data collected included horizontal activity for general health and gross motor skills; center time as an index of anxiety-related behavior (where less time spent in the center of the cage is interpreted as more anxiety-related behavior); and vertical activity as an index of depression-related behavior (where less vertical activity may indicate less escape behavior which is interpreted as more depression-related behavior) [33]–[37]. Locomotor activity was measured using an Omnitech Electronics Digiscan infrared photocell system [Test box model RXYZCM (16 TAO); Omnitech Electronics, Columbus, OH] as described before [38]. One hour activity measurements were obtained during animals' active cycle. Animals were placed singly in a 20×20×30 cm clear Plexiglas arena covered with a Plexiglas lid with multiple holes to ensure adequate ventilation. A photocell array measured horizontal locomotor activity using 8 pairs of infrared photocells located every 2.5 cm from side-to-side and 16 pairs of infrared photocells located front-to-back in a plane 2 cm above the floor of the arena. A second side-to-side array of 8 pairs of additional photocells located 5.5 cm above the arena floor measured vertical activity. Data were automatically gathered and transmitted to a computer via an Omnitech Model DCM-I-BBU analyzer.

### Acoustic startle reflex (ASR) test

Acoustic startle reflex is a characteristic sequence of involuntary, defensive, muscular responses elicited by a sudden, intense acoustic stimulus. Measurement of acoustic startle response with and without prepulse provides information about information processing and attention [39]–[43]. ASR was measured 3 days prior to injury (baseline; day −3) and on days 1, 14, and 30 post injury using a Med Associates Acoustic Response Test System (Med Associates, Georgia, VT) consisting of weight-sensitive platforms inside individual sound-attenuated chambers. Each mouse was placed individually in a ventilated holding cage that restricted extensive locomotion, but allowed them to turn around and make small movements. Animal movements in response to stimuli were measured as a voltage change by a strain gauge inside each platform. Responses were recorded by an interfaced Nexlink computer as the maximum response occurring during the no-stimulus periods, during the pre-pulse period, and during the startle period. Startle stimuli ranged from 100 to 110 dB and were white noise bursts of 20 msec duration sometimes preceded 100 msec by 68, 79, or 90 dB 1 kHz pure tones (pre-pulses). Each stimulus combination was presented six times. The total testing period was about 20 min.

### Behavior Data Analysis

Analysis of covariance (ANCOVA) and repeated measures analysis of covariance (rmANCOVA) were conducted for each of the behavioral variables, covarying for baseline scores to account for any baseline differences. NSS-R data were analyzed by a change score (1 day post injury – Baseline) [44]. OFL data were separated into three subscales: horizontal activity, center time, and vertical activity. The 100 dB ASR data were analyzed. All tests were two tailed using alpha  = .05. Data for all the behavior analysis is presented as mean ± standard error mean (SEM).

### MiRNA expression profiles in serum

To determine the effect of acute injury on the circulating miRNA expression profile, serum was collected via cardiac puncture at 3 hr post injury. 600–800 µl of blood was collected in the MiniCollect tube (Cat# 450472, Greiner Bio-One GmbH, Austria) and was allowed to clot at room temperature for 30–45 min. Serum was collected by centrifuging the blood samples at 3000 g/10 min at 4°C and immediately stored at −80°C. Total RNA enriched with miRNA was isolated from serum using miRNeasy Serum/Plasma Kit (Cat# 217184; Qiagen Inc. CA). MiRNA concentration was determined by Bioanalyzer (Agilent Technologies, Inc., Santa Clara, CA). Five ng of miRNA was taken for synthesizing complementary miRNA using the TaqMan MicroRNA Reverse Transcription (RT) Kit (Cat# 4366596; Life Technologies, Carlsbad, CA). For each sample, the following RT reaction mixture was set: 0.27 µl dNTP (100 M), 1 µl RT buffer (10X), 1.2 µl MgCl_2_ (25 mM), 0.14 µl RNase inhibitor (20 U/µl), 2 µl RT enzyme (50 U/µl), and 1 µl Megaplex RT Primers (either Rodent Primer Pool A [Cat# 4399826] or Rodent Primer Pool B V 3.0 [Cat# 4444299]). Five ng of miRNA in 3 µl volume was then mixed with the above 7 µl RT master mix to make a final reaction volume of 10 µl for each of the Pool A and B reaction. RT reaction was carried out on Veriti 96-Well Thermal Cycler (Life technologies, Carlsbad CA) as: [16°C/2 min; 42°C/1 min; 50°C/1 sec] X 40 cycles; 85°C/5 min; and hold at 4°C. Pre-amplification of the RT reaction was done as follows: Pre-amp master mix was made by mixing 12.5 µl TaqMan Pre-Amp Master Mix (2X) (Cat# 4384266); 2.5 µl of Megaplex Pre-Amp Rodent Primer (either Pool A [Cat# 4399922] or Pool B V 3.0 [Cat# 4444309]); and 5 µl nuclease free water. Five µl of RT reaction of either Pool A or Pool B was mixed with the 20 µl of the above respective Pre-amp master mix. Pre-amp reaction was carried out on Veriti 96-Well Thermal Cycler (Life technologies, Carlsbad CA) as: 95°C/10 min; 55°C/2 min; 72°C/2 min; [95°C/15 sec; 60°C/4 min] X 14 cycles; 99.9°C/10 min; and held at 4°C.

MiRNA profile was carried out using the TaqMan Rodent MicroRNA Array Set v3.0 (Life Technologies, Carlsbad, CA) as per manufacturer's protocol. Briefly, for each of the Pool A and B reactions, 9 µl of the pre-amp reaction product was mixed with 450 µl of TaqMan Universal Master Mix, no UNG (Cat# 4440040), and 441 µl of nuclease free water. Each of the reaction mixes was then loaded onto the respective pool's miRNA array plate. PCR reaction was carried out as 94.5°C/10 min; [97.0°C/30 sec; 59.7°C/1 min] X 40 cycles on 7900 HT Fast Real Time PCR machine (Life Technologies, Carlsbad, CA).

### Data analysis of miRNA array

MiRNA array threshold cycle (Ct) values were imported into the Real-Time StatMiner Software V.4.5.0.7 (Integromics, Madison, WI) for further analysis. StatMiner software allows merger of both plates A and B of the sample. Samples were grouped according to the weight and the fall height of the injury: IS1, IS2, IS3, IS4, and sham control. Imputation for missing values was skipped, and data were filtered for Ct <36 and “1 or more failure in 6 replicates”. Therefore, miRNAs that had Ct value <36 in all the six biological replicates were considered “expressed” and were taken for further analysis. Data were normalized using the “global normalization” as described elsewhere [45]. Relative quantitation (RQ) of the expression data was done over the sham controls and miRNAs that had more than 1.5 fold modulation with a *P value* <0.05 were considered as significantly modulated miRNAs. For the miRNAs where expression was absent (*i.e.*, Ct>36), the fold differences were calculated using the actual Ct value, with the maximum being 40. The “Ct status” is given as “calibrator not detected” or “target not detected” for expression being absent in “sham” or “injured” groups respectively. Ct status was given “valid” when the miRNA expression was detected (Ct<36) in both the “sham” and “injured” groups.

For the identification of miRNAs that may be used as “stably expressed miRNA” for validation of serum miRNA array data, the following approach was taken [46]: Ct values of all miRNAs across the samples, including both the injury and the sham groups, were taken. Mean and median Ct were calculated for each of the miRNA along with the standard deviation (SD). All miRNAs that had SD values >1 were eliminated. Remaining miRNAs were checked for similar average and median values and those differences greater than 0.5 were eliminated. Remaining miRNAs were checked for the mean Ct range of 14–20 to ensure abundant expression of the miRNA.

### Validation of individual miRNA expression

Expression of miRNAs, miR-214, miR-376a and miR-199a-3p was validated using the singleplex miRNA assay (Life technologies, Carlsbad, CA; Assay# 002306, 001069 and 002304, respectively). MiRNA miR-16 and miR-1937b were used as stable endogenous controls (Life Technologies, Carlsbad, CA; Assay# 000391 and 241023 respectively). A multiplex PCR was performed on 5 ng of miRNA as per manufacturer's protocol. A multiplex RT primer pool was prepared by mixing 10 µl of individual 5 X RT primers for each miRNA. 1X TE was added to make a final volume of 1000 µl. Multiplex RT reaction was set using the TaqMan MicroRNA Reverse Transcription (RT) Kit Cat# 4366596; Life Technologies, Carlsbad, CA) as follows: 6 µl of above multiplex RT primer pool; 0.3 µl of dNTP with dTTP (100 M); 3 µl of RT enzyme (50 U/µl); 1.5 µl of 10X RT buffer; 0.19 µl of RNAse inhibitor (20 U/µl); and 3 µl of RNA (5 ng). RT was carried out on Veriti 96-Well Thermal Cycler (Life technologies, Carlsbad CA) as follows: 16°C/30 min; 42°C/30 min; 85°C/5 min; and held at 4°C. For pre-amplification of RT product, multiplex pre-amplification primer pool was prepared by mixing 10 µl of individual 20X TaqMan assay primer for each miRNA. 1X TE was added to make a final volume of 1000 µl. Pre-amplification reaction was set as follows: 12.5 µl TaqMan Pre-Amp Master Mix (2X) (Cat# 4384266); 3.75 µl of above multiplex pre-amplification primer pool; 3.75 µl nuclease free water; and 5 µl of multiplex RT reaction product. Pre-amp reaction was carried out on Veriti 96-Well Thermal Cycler (Life technologies, Carlsbad CA) as: 95°C/10 min; 55°C/2 min; 72°C/2 min; [95°C/15 sec; 60°C/4 min] X 14 cycles; 99.9°C/10 min; and held at 4°C. Individual miRNA real time PCR was set in triplicates as follows: 10 µl of 2X TaqMan Universal Master Mix, no UNG (Cat# 4440040); 1 µl of 20X TaqMan Assay of miRNA; 7.67 µl nuclease free water; and 1.33 µl of multiplex pre-amp product (1∶5 diluted). Real Time PCR reaction was carried out as: 50°C/2 min; 95°C/10 min; [95°C/30 sec; 60°C/1 min] X 40 cycles on 7900 HT Fast Real Time PCR machine (Life Technologies, Carlsbad, CA). Three biological repeats were performed for each of the miRNAs and data are presented as fold change values using the comparative Ct method (2^−(mean ΔΔCt)^) values.

### Histology of brain sections

Following blood collection for the miRNA study, whole brains were harvested from mice and fixed in 10% normal buffered formalin. Fixed brain tissues were then processed, paraffin embedded, and 5 micron sections were cut at the hippocampus level and stained with H&E (Histoserv Inc., Germantown, MD). Sections were then evaluated under bright field microscopy.

## Results

### Mortality associated with the initial impact increased with the height of fall and rod weight

**Figure 1:**
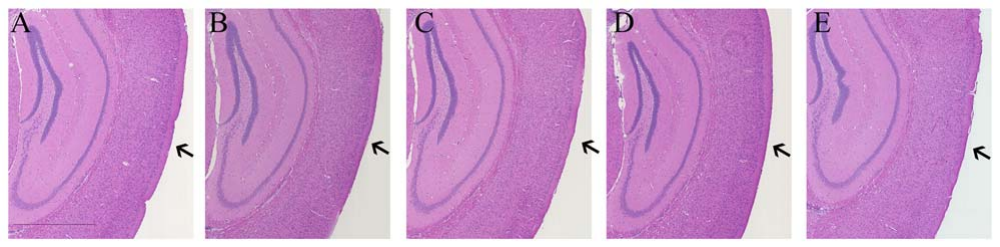
H&E stained sections of the brain. Histological evaluation of the brain tissue at the site of injury (arrow) was done to check for the lesion in the brain tissue following mTBI. No significant difference was observed in brain tissue sections between the sham (A), IS1 (B), IS2 (C), IS3 (D) and IS4 (E). Scale is 1 mm.

Animal mortality immediately after the impact with the falling weight increased with increase in the height of the fall and the weight of the rod. The percent mortality was 6.7±2.29, 41.3±5.4, 33.±14.11, and 65.9±11.5 in IS1, IS2, IS3, and IS4, respectively. No mortality was observed in the injured groups at the later time points in the experiment. Apnea immediately after the impact occurs in rodent model of weight drop injury and resuscitation with oxygen mask and gentle rubbing of the chest cavity improves recovery from the impact [25], [47]–[48]. The immediate mortality in the injury groups may have occurred due to the respiratory arrest upon impact [25]. The High mortality rate in our study as compared to the previously described similar model may be due to the slight modification to the impactor tip. Specifically, a silicon/resin covering was used in the previously described model [25], whereas no such covering was used in our system. H&E staining of the brain section at the hippocampus level (site of injury) did not reveal lesion volume in any of the injured groups, indicating mild brain injury in the animals that survived the initial impact of the injury (Figure 1).

### NSS-R scores increased with the increase in the fall height and weight

**Figure 2:**
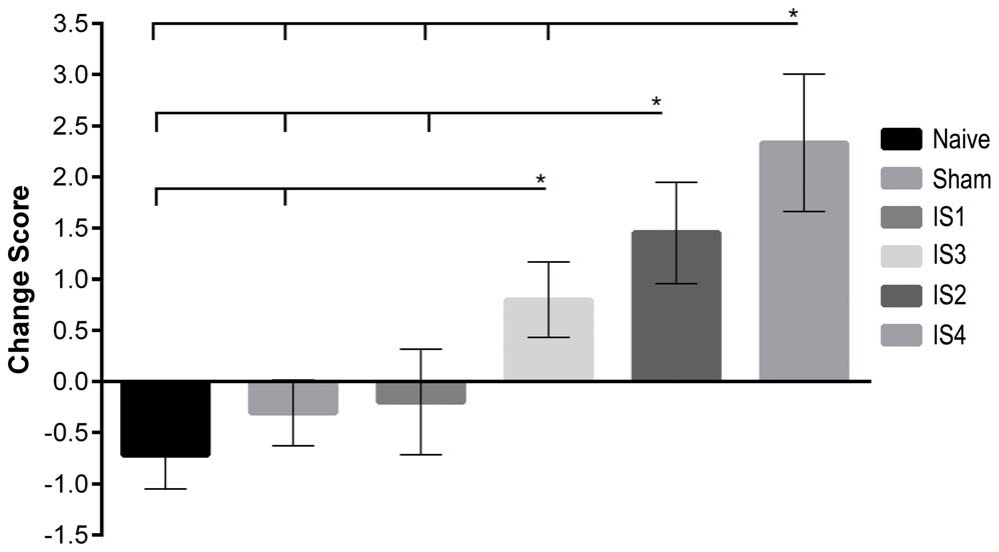
NSS-R for animals at day 1 post injury. Change scores between day 1 post injury and baseline were calculated. A gradual, but significant increase in the NSS-R scores were observed day 1 post injury with the increased severity of the injury within the mild spectrum. As expected, naïve and sham groups did not show any increase in their NSS-R scores. No change was observed in the IS1 group. NSS-R score of IS2, IS3, and IS4 groups increased and was the highest among the IS4 group. * *P value* <0.05.

NSS-R scores were determined by measuring behaviors of the naïve, sham, and injured animals in a series of 10 tasks to evaluate neurobehavioral effects of acute injury. NSS-R was measured at day −3 (baseline) and day 1 post injury. Significant increases in NSS-R change scores were observed with increasing severity of the injury (Figure 2). There were significant differences among injury groups, F(5,92) = 6.04, *P*<.001, η^2^ = .247. NSS-R change scores and group comparisons are presented in Table S2.

### Open field activity of the animals is reduced following the injury

**Figure 3:**
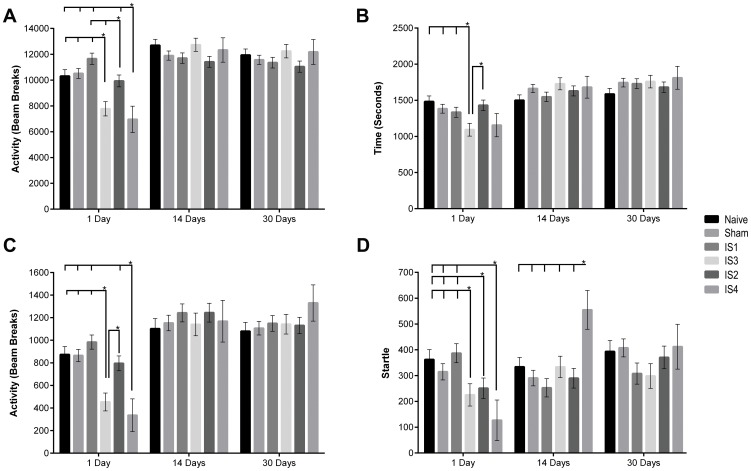
Neurobehavioral Activity. OFL test was conducted to evaluate animals' various activities in an open field as a measurement of behavioral deficits. Overall, day 1 showed significant reductions in the activities of injured animals as compared to the naïve and sham controls except for the IS1 group, which was not significantly different from that of naïve and sham groups. (**A**) Horizontal activity was evaluated as an indicator of the overall health. Activity was significantly reduced in the IS3 and IS4 groups. Activity in the IS2 group was significantly reduced as compared to the activity of IS1. Activity of IS1 and IS2 groups were not significantly different from the sham group. (**B**) Time spent in the center of the open field was evaluated as an indicator of anxiety-like behavior. Center time of the animals in IS3 and IS4 groups was reduced as compared to the 246 g injury groups (*i.e.*, IS1 and IS2), sham and naïve animals. The reduction in activity reached significance in the IS3. (**C**) Vertical activity was measured as an indicator of depression-related behavior. Vertical activity of the injured animals was significantly less than that of the sham and naïve animals except the IS1 group. * *P value* <0.05. (**D**) Startle response was measured as an indicator of emotional distress and potential sensory gating impairments. Startle responses of all injured groups, except the IS1 group, were reduced when compared to naïve and sham groups at day 1 post injury. There were no differences between groups at days 14 and 30 post injury, except in the IS4 group where the startle response was significantly higher than all other groups at day 14 post injury. Values are expressed as Mean ± SEM. * *P value* <0.05.

Horizontal activity of the animals was taken as a measure of the overall health of the animals prior to injury and after the injury. Activity was measured as the number of beam breaks during a 1 hr session. Overall, there was a significant Time x Injury interaction, F(8.33,245.14) = 7.69, *P*<.001, η^2^ = .207 (sphericity violated, used Greenhouse-Geisser correction). At 1 day post injury, there were significant differences among injury groups, F(5,149) = 8.46, *P*<.001, η^2^ = .221 (Figure 3A), such that the activity in IS2, IS3 and IS4 was reduced as compared to the naïve, sham, and IS1 groups. No significant differences were observed among the naïve, sham, and injury groups at 14 and 30 days post injury. Horizontal activity and comparisons among the groups appear in Table S3.

The time spent in the center of the open field was taken as a measurement of anxiety-related behavior. There was a significant effect of Time, F (2,294) = 16.26, *P*<.001, η^2^ = .100 (Table S4). There also was a significant Time × Injury interaction, F(10,294) = 3.45, *P*<.001, η^2^ = .105. At day 1 post injury, there were significant differences among injury groups, F(5,148) = 2.97, *P* = .014, η^2^ = .091 (Figure 3B), such that the center time was significantly reduced in the IS3 group as compared to the naïve, sham, IS1, and IS2 groups. At 14 and 30 days post injury, no significant differences between the control and injured groups were observed. Center time of each group and comparisons among groups appear in Table S5.

Vertical activity was taken as a measure of depression-related behavior such that less vertical activity is associated with more depression (*i.e.*, learned helplessness). Overall, there was a significant effect of Time, F(2,294) = 16.20, *P*<.001, η^2^ = .099 (Table S6). There was also a significant Time x Injury interaction, F(10,294) = 4.45, *P*<.001, η^2^ = .131 (Figure 3C). At 1 day post injury, there were significant differences among injury groups, F(5,148) = 7.32, p<.001, η2 = .198, such that the vertical activity in IS3 and IS4 groups was significantly reduced as compared to naïve, sham, IS1, and IS2 groups. No significant differences were observed between naïve, sham, and various injury groups at 14 and 30 days post injury. The vertical activity of each group and comparisons among groups appear in Table S7.

The overall negative effect on general health and vertical activity of the mouse was also evaluated as the percent of animals in injury groups that showed reduced activity compared to the naïve group at day 1 post injury. Each group was evaluated for the number of animals that showed activity less than the lower limit of the baseline activity of naïve animals. Overall, the more severe the injury, the greater percentage of the animals that showed reduced activity in the open field (Figure S2). To eliminate motor activity loss as a cause of reduced activity, animals were subjected to the rotarod test, which showed no significant differences between the various control and injury groups (Figure S3). No apparent signs of pain such as ruffled fur, hunched posture, altered behavior, vocalization, lethargy, tremors, ataxia *etc.* that may affect the activity of the injured mice were observed in the animals.

### Acoustic startle response was reduced in the animals following injury

Startle response of animals to a 100 dB tone was measured and is presented. Overall, when looking at the responses to a 100 dB tone alone, there was a significant Time x Injury interaction, F(10,264) = 3.72, *P*<.001, η^2^ = .123, such that a lower startle response was observed in the injured animals at day 1 post injury except in the IS4 group, and the response increased by day 14 and 30 post injury (Figure 3D). At day 1 post injury, there were significant differences among injury groups, F(5,132) = 3.54, *P* = .005, η^2^ = .118 (Table S8). On day 14 post injury, there were significant differences among injury groups, F(5,132) = 2.95, *P* = .015, η^2^ = .100 (Table S9). No significant differences were observed between naïve, sham, and various injury groups at 30 days post injury.

### Serum miRNAs are modulated in injured animals

**Figure 4:**
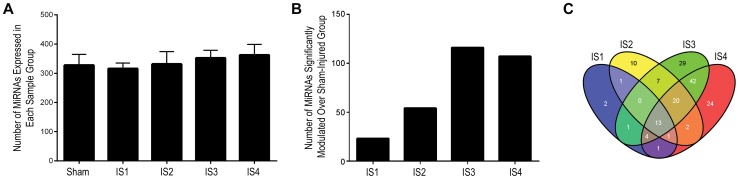
MiRNA expression pattern. (**A**) MiRNAs that show Ct<36 were considered as expressed. The number of miRNAs expressed ranged from 315.83−362.17 with a maximum and minimum numbers detected in IS4 and IS1 respectively. The SD ranged from 19.28−43.37, the maximum being in IS2 and the minimum in IS1. The values are presented as Mean ± SD of the number of miRNAs detected in each group. (B) The number of miRNAs that were significantly modulated (≥1.5 fold; *P*<0.05) following the injury over the time point matched sham controls increased with the height and weight of the free falling metal rod except in the IS3 group where the number of significantly modulated miRNAs was marginally greater than the IS4 group. (C) Overlapping miRNA data analysis for significantly modulated miRNAs in the injury groups was done using the online Venn diagram generation tool [Oliveros (2007). VENNY. An interactive tool for comparing lists with Venn Diagrams. http://bioinfogp.cnb.csic.es/tools/venny/index.html].

To determine whether miRNAs were modulated after the injury, serum miRNA expression of the injured mice was compared to the serum miRNA expression of the sham mice at 3 hr post injury. The numbers of the miRNA that passed the detection criteria were similar among the injured and sham groups (Figure 4A). However, the number of significantly modulated miRNAs increased with the increasing grade of injury except in the IS4 injury group where the numbers of significantly modulated miRNA were marginally less than the IS3 injury group (Figure 4B). 23 miRNAs (14 up- and 9 down- regulated) were significantly modulated in IS1 injury group (Table 1). 53 miRNAs (35 up- and 18 down-regulated) were significantly modulated in IS2 injury group (Table 2). 116 miRNAs (70 up- and 46 down-regulated) were significantly modulated in IS3 injury group (Table 3). 106 miRNAs (66 up- and 40 down-regulated) were significantly modulated in IS4 injury group (Table 4). Hierarchical clustering of the DDCt values of the expressed miRNAs showed IS3 and IS4 (*i.e.*, 333 g groups) were similar to each other followed by the association with the IS2. The IS1 group clustered away from the rest of the three injury groups (Figure S4). This observation was consistent with the behavior data where the IS1 group did not show any significant differences from the sham group.

**Table 1:** Significantly modulated miRNA in IS1.

S. No.	MiRNA	P Value	Fold Difference(log10 [RQ])
**Calibrator not detected**			
1	mmu-miR-376a	9.43E-04	1.99
2	mmu-miR-494	1.85E-04	1.94
3	mmu-miR-297a#	3.11E-02	1.56
4	rno-miR-345-3p	4.78E-03	1.08
**Valid**			
5	hsa-miR-214	1.82E-03	0.58
6	mmu-miR-214	6.37E-03	0.54
7	mmu-miR-337-5p	2.10E-02	0.52
8	mmu-miR-574-3p	2.16E-02	0.51
9	mmu-miR-434-3p	2.16E-02	0.41
10	mmu-miR-671-3p	1.76E-02	0.39
11	mmu-miR-218	4.46E-02	0.36
12	mmu-miR-676	1.58E-02	0.33
13	mmu-miR-199a-3p	3.34E-02	0.32
14	hsa-miR-455	4.71E-02	0.28
15	mmu-miR-322	9.85E-03	−0.34
16	mmu-miR-331-3p	1.99E-02	−0.35
17	hsa-miR-106b#	2.80E-02	−0.40
18	mmu-miR-106b	1.41E-02	−0.47
19	mmu-miR-2138	5.83E-03	−0.61
20	mmu-miR-31	2.16E-02	−0.85
**Target not detected**			
21	mmu-miR-363	4.59E-02	−1.40
22	mmu-miR-181c	2.86E-02	−1.66
23	rno-miR-196c	4.66E-02	−1.81

Twenty three miRNA were significantly modulated. Of these 14 were up regulated and 9 were down regulated. Values are given as Log10 of the fold change (2^−(mean ΔΔCt)^).

**Table 2:** Significantly modulated miRNA in IS2.

S.No.	MiRNA	P Value	Fold Difference (log10 [RQ])
**Calibrator not detected**			
1	mmu-miR-700	2.31E-02	2.21
2	mmu-miR-487b	2.17E-03	1.91
3	mmu-miR-376a	1.11E-03	1.70
4	mmu-miR-125b	4.32E-02	1.23
5	mmu-miR-1932	2.25E-02	1.17
6	mmu-miR-218-1	3.42E-02	1.05
7	mmu-miR-191*	1.79E-02	1.00
**Valid**			
8	mmu-miR-384-5p	6.70E-03	0.73
9	hsa-miR-214	2.58E-05	0.72
10	mmu-miR-667	5.12E-03	0.69
11	mmu-miR-218	1.82E-04	0.64
12	mmu-miR-214	8.97E-05	0.64
13	mmu-miR-434-3p	3.91E-03	0.61
14	mmu-miR-122	1.59E-03	0.61
15	mmu-miR-34b-3p	3.05E-03	0.60
16	mmu-miR-872*	9.36E-03	0.56
17	mmu-miR-361	8.35E-03	0.55
18	mmu-miR-485-3p	2.64E-02	0.53
19	mmu-miR-574-3p	2.61E-03	0.53
20	hsa-miR-9*	4.56E-02	0.52
21	mmu-miR-337-5p	9.90E-03	0.49
22	mmu-miR-685	3.22E-03	0.48
23	mmu-miR-204	1.44E-02	0.44
24	mmu-miR-376c	2.57E-02	0.43
25	mmu-miR-199a-3p	2.91E-03	0.42
26	mmu-miR-337-3p	2.11E-02	0.40
27	mmu-miR-132	4.27E-02	0.40
28	mmu-miR-671-3p	1.04E-02	0.37
29	mmu-miR-152	1.58E-02	0.36
30	mmu-miR-376b*	1.29E-02	0.34
31	mmu-miR-212	3.85E-02	0.32
32	mmu-miR-511	1.48E-02	0.31
33	mmu-miR-192	2.19E-03	0.30
34	mmu-miR-145	2.81E-02	0.25
35	mmu-miR-146a	3.56E-02	0.24
36	mmu-miR-19b	3.57E-02	−0.25
37	mmu-miR-322	3.22E-02	−0.26
38	hsa-miR-200c	1.20E-02	−0.26
39	mmu-miR-486	2.56E-02	−0.27
40	mmu-miR-1274a	3.52E-02	−0.28
41	hsa-miR-200b	4.62E-02	−0.30
42	mmu-miR-2146	2.90E-02	−0.30
43	mmu-miR-652	1.91E-02	−0.30
44	mmu-miR-18a	2.17E-02	−0.34
45	hsa-miR-106b*	9.68E-03	−0.38
46	mmu-miR-451	1.48E-02	−0.42
47	mmu-miR-106b	7.93E-03	−0.45
48	mmu-miR-26b	3.19E-02	−0.53
49	mmu-miR-31	1.86E-02	−1.03
50	hsa-miR-875-5p	4.14E-02	−3.02
**Target not detected**			
51	rno-miR-196c	4.67E-02	−1.53
52	mmu-miR-1982.2	3.95E-02	−1.56
53	mmu-miR-7a	8.20E-03	−1.98

Fifty three miRNA were significantly modulated. Of these, 35 were up regulated and 18 were down regulated. Values are given as Log10 of the fold change (2^−(mean ΔΔCt)^).

**Table 3:** Significantly modulated miRNA in IS3.

S.No	MiRNA	P Value	Fold Difference (log10 [RQ])
**Calibrator not detected**			
1	mmu-miR-15a*	1.8E-13	4.57
2	mmu-miR-495	1.5E-03	2.87
3	mmu-miR-673	3.0E-02	2.66
4	mmu-miR-433	9.8E-04	2.63
5	mmu-miR-146b	2.7E-02	2.48
6	mmu-miR-702	2.4E-02	2.24
7	mmu-miR-10a	2.6E-02	2.22
8	mmu-miR-700	2.4E-02	2.2
9	mmu-miR-134	1.9E-02	2.09
10	mmu-miR-487b	1.3E-03	2.01
11	mmu-miR-494	7.3E-05	1.96
12	hsa-miR-99b*	7.7E-03	1.87
13	mmu-miR-551b	2.1E-03	1.75
14	mmu-miR-376a	7.1E-04	1.72
15	rno-miR-551B	5.7E-03	1.62
16	mmu-miR-434-5p	2.9E-02	1.61
17	rno-miR-204*	1.7E-02	1.6
18	mmu-miR-467b	1.6E-02	1.6
19	mmu-miR-137	2.7E-02	1.54
20	rno-miR-345-3p	2.0E-03	1.41
21	mmu-miR-547	4.1E-02	1.3
22	mmu-miR-680	4.8E-02	1.14
23	mmu-miR-218-1*	3.0E-02	1.1
24	mmu-miR-345-3p	3.4E-02	1.09
25	mmu-miR-295	2.6E-02	1.09
26	rno-miR-351	6.3E-03	0.89
**Valid**			
27	mmu-miR-122	2.2E-04	1.24
28	mmu-miR-872	2.6E-02	0.98
29	mmu-miR-667	7.9E-04	0.91
30	mmu-miR-214	2.4E-07	0.88
31	mmu-miR-485-3p	5.1E-04	0.83
32	mmu-miR-365	9.5E-03	0.83
33	mmu-miR-384-5p	9.8E-04	0.78
34	mmu-miR-337-5p	1.0E-05	0.77
35	mmu-miR-193*	7.4E-03	0.74
36	mmu-miR-671-3p	2.1E-05	0.72
37	hsa-miR-214	2.5E-05	0.71
38	mmu-miR-574-3p	4.2E-05	0.71
39	mmu-miR-192	3.5E-03	0.64
40	mmu-miR-434-3p	7.2E-05	0.64
41	mmu-miR-685	3.1E-04	0.63
42	mmu-miR-193	3.5E-02	0.62
43	mmu-miR-872*	2.8E-03	0.6
44	mmu-let-7a*	4.4E-03	0.6
45	mmu-miR-410	4.8E-03	0.58
46	mmu-miR-125b-5p	1.6E-02	0.57
47	mmu-miR-218	1.3E-03	0.54
48	mmu-miR-34b-3p	2.5E-04	0.54
49	mmu-miR-145	1.2E-03	0.53
50	mmu-miR-500	8.2E-05	0.51
51	mmu-miR-132	6.0E-03	0.48
52	mmu-miR-127	3.3E-02	0.46
53	mmu-miR-125a-5p	3.1E-03	0.46
54	mmu-miR-199a-3p	1.4E-03	0.46
55	mmu-miR-706	2.0E-02	0.44
56	hsa-miR-671-5p	1.8E-02	0.44
57	mmu-miR-152	2.4E-03	0.44
58	mmu-miR-1944	4.0E-02	0.42
59	mmu-miR-361	2.7E-02	0.38
60	mmu-miR-376c	8.4E-03	0.37
61	mmu-miR-362-3p	4.0E-02	0.37
62	hsa-miR-744*	7.7E-03	0.36
63	hsa-miR-455	3.0E-02	0.35
64	mmu-miR-31*	2.4E-02	0.35
65	mmu-miR-674*	4.3E-02	0.34
66	mmu-miR-204	4.2E-03	0.32
67	mmu-miR-212	2.4E-02	0.29
68	mmu-miR-532-3p	2.9E-02	0.28
69	mmu-miR-511	2.8E-02	0.26
70	mmu-miR-1839-3p	4.9E-02	0.19
71	mmu-miR-200c	4.7E-02	−0.22
72	mmu-miR-484	2.7E-02	−0.23
73	mmu-miR-18a*	1.6E-03	−0.25
74	hsa-miR-425	4.7E-02	−0.26
75	mmu-miR-1930	3.6E-02	−0.26
76	mmu-miR-301a	4.2E-02	−0.26
77	mmu-miR-2134	3.9E-03	−0.28
78	mmu-miR-19b	1.3E-02	−0.29
79	mmu-miR-222	4.6E-02	−0.31
80	mmu-miR-141	3.9E-02	−0.33
81	hsa-miR-200c	2.1E-03	−0.33
82	mmu-miR-106a	2.0E-02	−0.34
83	mmu-miR-130b	5.5E-03	−0.34
84	mmu-miR-486	1.7E-02	−0.35
85	mmu-miR-17	9.6E-03	−0.39
86	mmu-miR-331-3p	1.3E-02	−0.41
87	mmu-miR-652	7.1E-03	−0.41
88	mmu-let-7d	7.6E-03	−0.41
89	mmu-miR-17*	9.6E-03	−0.43
90	mmu-miR-16	2.7E-02	−0.43
91	hsa-miR-421	6.9E-04	−0.43
92	mmu-miR-345-5p	2.4E-03	−0.43
93	mmu-miR-18a	1.6E-03	−0.43
94	mmu-miR-103	2.1E-02	−0.44
95	mmu-miR-25	4.3E-03	−0.47
96	mmu-miR-93	4.8E-03	−0.47
97	mmu-miR-140	1.7E-02	−0.48
98	mmu-let-7i	1.5E-03	−0.48
99	mmu-miR-20a	8.0E-04	−0.48
100	mmu-miR-20b	2.5E-02	−0.49
101	mmu-miR-340-5p	2.0E-02	−0.5
102	mmu-miR-186*	1.6E-02	−0.51
103	mmu-miR-301b	7.0E-03	−0.54
104	mmu-miR-15b	5.0E-04	−0.54
105	mmu-miR-26a	4.1E-03	−0.57
106	mmu-miR-142-3p	4.2E-03	−0.6
107	mmu-miR-26b	7.4E-03	−0.63
108	hsa-miR-106b*	5.9E-05	−0.64
109	mmu-miR-451	5.0E-05	−0.78
110	mmu-miR-106b	4.2E-05	−0.83
111	mmu-miR-31	2.4E-02	−0.87
112	hsa-miR-875-5p	2.5E-02	−3.16
**Target not detected**			
113	rno-miR-196c	2.2E-02	−1.39
114	mmu-miR-463	2.2E-03	−1.75
115	mmu-let-7e	3.7E-02	−1.98
116	mmu-miR-363	4.4E-03	−2.95

One hundred and sixteen miRNA were significantly modulated. Of these, 70 were up regulated and 46 were down regulated. Values are given as Log10 of the fold change (2^−(mean ΔΔCt)^).

**Table 4:** Significantly modulated miRNA in IS4.

S. No.	MiRNA	P Value	Fold Difference (log10 [RQ])
**Calibrator not detected**			
1	mmu-miR-15a*	2.14E-13	4.47
2	mmu-miR-129-3p	6.98E-05	3.37
3	hsa-miR-149	7.43E-03	3.33
4	mmu-miR-187	1.92E-03	3.22
5	mmu-miR-433	7.82E-04	2.87
6	mmu-miR-34c	1.08E-04	2.81
7	mmu-miR-146b	2.44E-02	2.52
8	hsa-let-7e*	3.67E-07	2.27
9	mmu-miR-10a	2.49E-02	2.23
10	mmu-miR-134	2.06E-02	2.07
11	mmu-miR-494	1.42E-04	2.02
12	rno-miR-204*	5.97E-03	1.97
13	mmu-miR-383	3.11E-03	1.89
14	mmu-miR-487b	2.96E-03	1.87
15	mmu-miR-376a	4.14E-03	1.68
16	mmu-miR-137	2.01E-02	1.65
17	rno-miR-345-3p	5.67E-04	1.61
18	mmu-miR-381	3.45E-03	1.60
19	mmu-miR-455	3.32E-02	1.59
20	hsa-miR-99b*	1.94E-02	1.58
21	mmu-miR-125b*	3.70E-02	1.28
22	mmu-miR-345-3p	2.48E-02	1.17
23	mmu-miR-218-1*	4.93E-02	0.98
24	rno-miR-351	8.86E-03	0.86
**Valid**			
25	mmu-miR-122	5.56E-05	1.41
26	mmu-miR-667	9.46E-04	1.19
27	mmu-miR-365	2.39E-03	0.93
28	mmu-miR-34b-3p	6.71E-04	0.91
29	mmu-miR-485-3p	3.61E-04	0.84
30	mmu-miR-214	4.42E-07	0.80
31	mmu-miR-204	1.71E-04	0.77
32	hsa-miR-214	1.03E-05	0.75
33	mmu-miR-193*	1.37E-03	0.74
34	mmu-miR-384-5p	2.60E-02	0.74
35	mmu-miR-125b-5p	2.47E-03	0.70
36	mmu-miR-194	1.78E-03	0.69
37	mmu-miR-127	1.59E-02	0.69
38	mmu-miR-671-3p	1.94E-04	0.69
39	mmu-miR-434-3p	1.61E-03	0.67
40	mmu-miR-199a-3p	1.01E-04	0.62
41	mmu-miR-132	6.76E-03	0.61
42	mmu-miR-410	2.09E-02	0.60
43	mmu-miR-872*	8.59E-03	0.58
44	mmu-miR-192	3.06E-04	0.57
45	mmu-miR-145	1.40E-03	0.56
46	hsa-miR-671-5p	4.68E-03	0.53
47	mmu-miR-574-3p	1.26E-03	0.53
48	mmu-miR-532-5p	1.23E-02	0.52
49	mmu-miR-337-5p	5.90E-03	0.52
50	mmu-miR-375	4.70E-02	0.50
51	mmu-miR-218	7.99E-04	0.50
52	mmu-let-7a*	5.98E-03	0.49
53	hsa-miR-455	2.99E-03	0.49
54	mmu-miR-500	2.66E-03	0.46
55	mmu-miR-1944	1.63E-02	0.45
56	mmu-miR-685	1.30E-03	0.43
57	mmu-miR-148a	1.47E-03	0.42
58	mmu-miR-212	3.16E-02	0.41
59	mmu-miR-224	6.52E-03	0.40
60	mmu-miR-676	2.96E-03	0.40
61	mmu-miR-152	9.64E-03	0.40
62	mmu-miR-200a	4.06E-02	0.39
63	hsa-miR-744*	1.19E-02	0.36
64	mmu-miR-497	9.60E-03	0.35
65	mmu-miR-532-3p	2.04E-02	0.30
66	mmu-miR-133a	4.79E-02	0.27
67	mmu-miR-19b	2.20E-02	−0.27
68	mmu-miR-28	1.19E-02	−0.27
69	mmu-miR-18a*	1.54E-02	−0.30
70	mmu-miR-301a	2.62E-02	−0.32
71	mmu-miR-18a	2.96E-02	−0.32
72	mmu-miR-15b	7.79E-03	−0.34
73	mmu-miR-879*	4.04E-02	−0.35
74	mmu-miR-191	2.35E-03	−0.35
75	mmu-let-7i	2.49E-02	−0.35
76	mmu-miR-222	1.42E-02	−0.35
77	mmu-let-7d	1.78E-02	−0.36
78	hsa-miR-106b*	1.21E-02	−0.36
79	mmu-miR-93	2.02E-02	−0.36
80	mmu-miR-106a	2.31E-02	−0.37
81	mmu-miR-16*	1.54E-02	−0.39
82	mmu-miR-25	3.56E-02	−0.39
83	mmu-miR-130b	2.38E-02	−0.40
84	mmu-miR-2134	2.10E-02	−0.41
85	mmu-miR-17	4.15E-03	−0.43
86	mmu-miR-16	3.14E-02	−0.43
87	mmu-miR-15a	2.51E-02	−0.44
88	rno-miR-148b-5p	1.10E-02	−0.45
89	mmu-miR-451	1.04E-02	−0.51
90	mmu-miR-340-5p	1.11E-02	−0.53
91	mmu-miR-20a	3.68E-03	−0.53
92	hsa-miR-421	5.54E-04	−0.53
93	mmu-miR-26b	1.48E-02	−0.57
94	mmu-miR-1274a	2.25E-02	−0.60
95	mmu-miR-186	3.50E-02	−0.60
96	mmu-miR-142-3p	4.12E-03	−0.61
97	mmu-miR-17*	4.59E-03	−0.62
98	mmu-miR-301b	2.02E-03	−0.66
99	mmu-miR-26a	1.46E-03	−0.66
100	mmu-miR-186*	2.64E-03	−0.67
101	mmu-miR-106b	1.92E-03	−0.68
102	mmu-miR-31	1.59E-02	−0.86
103	hsa-miR-875-5p	1.02E-02	−3.75
**Target not detected**			
104	rno-miR-196c	4.12E-02	−1.80
105	mmu-miR-363	1.33E-02	−2.38
106	mmu-miR-2138	4.71E-02	−2.52

One hundred and six miRNA were significantly modulated. Of these, 66 were up regulated and 40 were down regulated. Values are given as Log10 of the fold change (2^−(mean ΔΔCt)^).

### Common miRNA changes regardless of the mTBI severity

To identify miRNAs that may consistently be present regardless of extent or severity of the injury, significantly modulated miRNAs were compared among the injury groups (Figure 4C). 13 miRNAs were found to be modulated in all four injury groups and had similar expression patterns. Nine of the 13 miRNAs were up regulated and 4 were down regulated following injury (Table 5). The Ct values of the modulated miRNAs are given in Table S10.

**Table 5:** Common MiRNA among all injury groups.

S. No.	MiRNA	IS1	IS2	IS3	IS4
1	mmu-miR-376a	1.99	1.70	1.72	1.68
2	hsa-miR-214	0.58	0.72	0.71	0.75
3	mmu-miR-214	0.54	0.64	0.88	0.80
4	mmu-miR-337-5p	0.52	0.49	0.77	0.52
5	mmu-miR-574-3p	0.51	0.53	0.71	0.53
6	mmu-miR-434-3p	0.41	0.61	0.64	0.67
7	mmu-miR-671-3p	0.39	0.37	0.72	0.69
8	mmu-miR-218	0.36	0.64	0.54	0.50
9	mmu-miR-199a-3p	0.32	0.42	0.46	0.62
10	hsa-miR-106b*	−0.40	−0.38	−0.64	−0.36
11	mmu-miR-106b	−0.47	−0.45	−0.83	−0.68
12	mmu-miR-31	−0.85	−1.03	−0.87	−0.86
13	rno-miR-196c	−1.81	−1.53	−1.39	−1.80

Thirteen miRNAs were commonly modulated among all the injury groups. Of these, 9 were up regulated and 4 were down regulated. Values are given as Log10 of the fold change (2^−(mean ΔΔCt)^).

### Brain function specific miRNAs are modulated following mTBI

**Figure 5:**
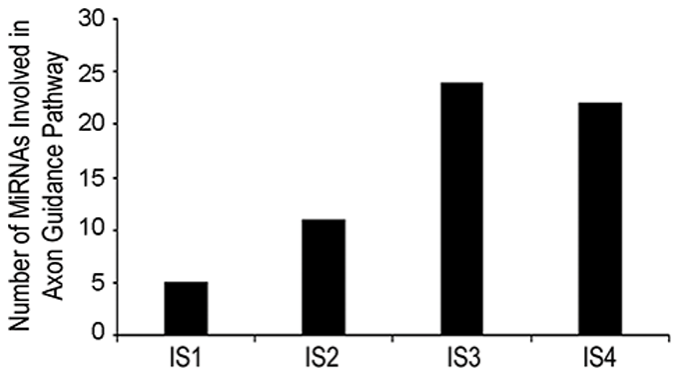
Involvement of significantly modulated miRNA in axon guidance pathway. Quantitative bioinformatics analysis on the number of miRNA involved in the axon guidance pathway was done using IPA. The number of the significantly modulated miRNAs that were predicted to modulate axon guidance pathway increased with the severity of the injury.

The miRNAs common among all mTBI groups were analyzed for their combined effect on functional pathways using the DIANA-miRPath v2.0 [49]. Kyoto Encyclopedia of Genes and Genomes (KEGG) pathways that were most significantly predicted to be affected by the combined effect of the modulated miRNAs were identified. Several brain function related pathways, such as axon guidance, gap junction, dopaminergic synapse, cholinergic synapse, long-term potentiation, tight junction, adherens junction, and long-term depression, were found to be targeted by the modulated miRNAs (Figure S5). Specific analysis of the axon guidance pathway, previously reported as affected upon TBI, showed that the number of the miRNAs predicted to be involved in this pathway increased with the severity of the injury (Figure 5). The combined effect of modulated miRNAs on functional pathways using the DIANA-miRPath v2.0 [49] was also performed for the significantly modulated miRNAs common among the three injury groups that showed significant change in NSS-R, OFL, and ASR tests (*i.e.*, IS2, IS3, and IS4) (Table S11). The axon guidance pathway, along with several other brain related function pathways, was one of the most affected KEGG Pathway (Figure S6). Unique miRNAs that were modulated only in the IS1 group (Table S12), which did not show any significantly difference in NSS-R and OFL tests, showed long-term potentiation pathways as the most affected pathway by DIANA-miRPath v2.0 analysis (data not shown).

**Figure 6:**
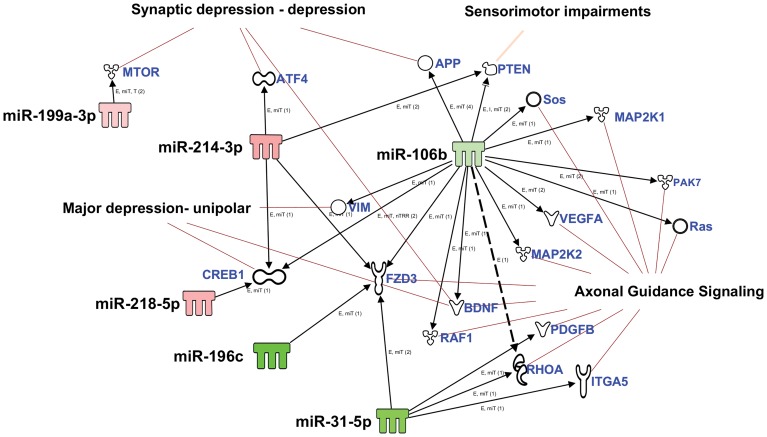
IPA Analysis for effect of significantly modulated miRNAs common among all four injury groups on brain functions related pathways. MiRNAs common to all four injury groups were taken and their targets and pathway analysis in relation to brain related functions were performed. My Pathway tool of IPA software was used to build the custom pathway using miRNAs that show experimentally validated targets by overlaying with 1) depression, 2) sensorimotor function and the 3) axon guidance pathway molecules. Of the 13 common modulated miRNAs, only 6 miRNAs were found to have experimentally validated targets that are predicted to modulate axon guidance, synaptic depression and sensorimotor impairments.

**Figure 7:**
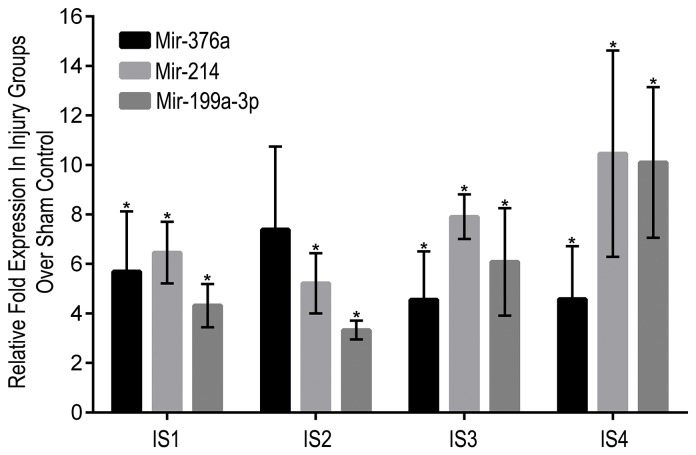
Expression of miRNAs in individual real time PCR assay. The fold upregulation of three miRNAs, miR-376a, miR-214 and miR-199a-3p, in the injury groups over the sham mice was validated using the individual real time PCR assays. Similar to the miRNA arrays, expression of the three miRNAs was found to be up regulated in the injured groups over the sham animals. Data presented is the fold up regulation (± SEM; *P<0.05) calculated from the mean DDCt value obtained from three biological samples for each injury group. Statistical significance was calculated using the individual Ct values obtained from the three biological replicates for each injury group.

Correlation analysis of the modulated miRNAs and their validated targets by the IPA software revealed a direct relation with validated targets of six of the modulated miRNAs to the axon guidance pathway (Figure 6). Because behavioral analysis showed symptoms of depression in the injured animals, a correlation analysis of the modulated miRNAs with depression-related pathways was performed, this showed their direct effect on the depression-related validated targets (Figure 6). The molecular pathway for sensorimotor impairments was also predicted to be targeted by the significantly modulated miRNAs (Figure 6). This correlates with the NSS-R and ASR measurements that also showed sensorimotor impairments. The expression profile of 3 out of 13 commonly modulated miRNAs, miR-199-3p, miR-214, and miR-376a that have been shown to be important in brain related processes and functions was also validated using the singleplex miRNA assay (Life Technologies, Carlsbad, CA) (Figure 7).

### Stable miRNA expression in serum

To identify miRNAs that can be used for validating the expression data of the miRNA array, miRNAs that exhibited stable expression in the serum, irrespective of the sham or injury group, were identified. 18 miRNAs were identified that showed stable Ct values across the sham and injury groups with SD <1 (Table S13). One of the miRNAs (miR-16) has been described as stable serum miRNA in other studies [50]. Therefore, miR-16 (Mean Ct  = 15.54±0.91) along with another miRNAs, miR-1937b (Mean Ct  = 14.91±0.90) were taken for use as endogenous controls in the miRNA expression validation experiments. Similar results were obtained with either of the selected stable miRNAs (data not shown) and data presented here used miR-16 as the stable endogenous miRNA. MiR-1937b is no longer considered a miRNA as it is a tRNA-derived small RNA fragment (tRF) (http://www.mirbase.org/cgi-bin/mirna_entry.pl?acc=MI0009938). Therefore, miR-1937b (like non-coding small nuclear RNA U6), presents a target that is of non-miRNA origin and may be useful as stable endogenous small RNA to validate miRNA expression in serum.

## Discussion

MTBI is a heterogeneous injury that can range from no clinical symptoms to development of post concussive syndrome (PCS) after injury. Rapp and Curly [51] describe mTBI as an event that may lead to the development of neurological disorders. The heterogeneous nature of mTBI makes it difficult to diagnose, based exclusively on current behavioral and neurocognitive analyses [51]. Biochemical assays for brain related proteins in serum, such as GFAP, UCH-L1 and S100β, are either not sensitive or selective for mTBI [7]–[12]. Therefore, a more reliable and sensitive molecular assay is needed to accurately diagnose mTBI. In this experiment, acute phase miRNA changes in the serum were measured to determine the feasibility of using miRNA as diagnostic markers of mTBI.

Increasing grades of mTBI were induced by a weight drop device by increasing the height and weight of the falling rod. Injuries were characterized by the sensory motor responses designed to model clinical neurological examination and indicated the injuries to be within the mild spectrum of TBI [26]–[27]. Consistent with mTBI, histological analysis of the brain showed no gross pathological changes following the injury. Behavior impairments such as depression and loss of interest have been described in patients with mTBI [52]. Reduced escape behavior, measured in terms of vertical activity of the animal in an open field apparatus, can be a measure of depression-related behavior [53]–[55]. Transient, but significant depression-related behavior in terms of reduced vertical activity was observed in the injured animals at day 1 post injury, which recovered over time. A general negative impact on the overall health of the animals was also observed at day 1 post injury, indicated by the reduction in horizontal activity of the injured animals. Sensory gating impairments have also been reported in mTBI [56]. Increase in the OFL activity of the animals at the later time points of day 14 and day 30 as compared to day 1 activity was interesting. We hypothesize that the naïve and sham control animals remember being in the chambers and that the other animals, due to injury, may not remember the chamber as well and spend more time exploring. ASR is a complex behavior that engages higher brain functions and is related to information processing [39], [41], [57]–[58]. A significantly reduced startle response was observed in animals with injury, such that the greater the injury severity, the less startle response was observed. The parietal lobe, where the injury was centered, is involved in the sensorimotor information processing [59]. It is suggested that the right inferior parietal lobe plays a role in auditory signal processing and integration of sensory and motor functions [39], [59]–[60]. This observation indicates the diffuse nature of the injury that may also affect central brain functions. Many individuals, who suffer with mTBI do not exhibit neurobehavioral alterations and acute symptoms of mTBI, such as headaches, sleep disturbance and depression or recover spontaneously from the injury [13]–[15]. A similar observation was made in the current experiment, where the percent of animals' OFL activity in the mildest of mild injury group (*i.e.*, IS1) was similar to sham and naïve control groups. Other groups, with a more severe form of the mild injury, however, showed greater reduction in the percent activity than the sham and naïve groups.

MiRNA changes in the serum have been suggested as a potential marker of disease and injury [16]–[18]. MiRNA modulations in the serum of TBI patients have been reported. Down regulated expression of has-mir-16, and has-mir-92 and increased levels of has-mir-765 correlated with the severe TBI, however, their utility in diagnosing mTBI was limited [24]. Earlier we have also reported the modulation of let-7i in the serum and cerebrospinal fluid of rats following blast injury [22]. Modulation of miRNA expression in the brain following TBI has been shown before [19]-[23]. Some of the miRNAs (mir-21, mir-34a, mir-27b, mir-9, mir-874, mir-223, mir-144 and mir-153) that were reported to increase in the brain after moderate to severe TBI [20]–[21], [23], [61]–[62] did not show significant modulation in the serum in our experiment. Mir-497 and mir-149, which were up regulated in IS4 group, were earlier reported to also increase in the brain after TBI [23], [63]. Mir-451 and mir-340-5p, which were shown to be up regulated in the brain post TBI, were down regulated in our experiment [19], [61]. Despite the difference in the tissue being analyzed after the TBI, some common targets of modulated miRNAs were observed. Earlier studies found apoptosis as one of the cellular process targeted by the miRNAs modulated in the brain following TBI [19]–[21]. Apoptosis was predicted to be affected by the modulated miRNAs in this experiment as well. BDNF, which was identified as a predicted target of one of the down regulated miRNA mir-106 in this experiment, was also identified as target of the modulated miRNAs following TBI in an earlier study [19].

Two main observations were made in the current experiment. First, the number of miRNAs that were significantly modulated post injury increased with the severity of the injury. Second, thirteen common miRNAs were significantly modulated in all the four injury groups compared to the sham controls. Functional analysis to identify the combined effect of modulated miRNAs showed that eight of the thirteen miRNAs may play a role in CNS related pathways, such as synaptic depression, sensorimotor impairments and the axon guidance pathway (Figure 5, Figure S5). Vimentin (VIM) and phosphatase and tensin homolog (PTEN), targets of the significantly down regulated miRNA mir-106b, expression has been shown to increase in the brain post TBI and has been related to inflammatory cell proliferation and differentiation and neuronal survival and neurite integrity, respectively [64]–[67]. Brain-derived neurotrophic factor (BDNF) and Amyloid precursor protein (APP), also targets of mir-106b, have been shown to increase post TBI [68]–[69]. BDNF has been reported to have a neuroprotective effect post TBI [68], [70]. The role of APP is, however, debated as some studies have shown APP association with neuronal loss and others have shown APP as neuroprotective [71]–[74]. Axon guidance includes axon outgrowth, repulsion, and attraction, which plays an important role in neuronal functions and axonal regeneration. Axonal injury is prevalent in TBI [75]–[78]. Axon guidance and synaptic plasticity is affected post TBI and positive regulation of axon guidance has been suggested to result in better functional outcomes [79]–[81]. Elevated levels of axon related proteins, such as neurofilament heavy chain, Tau, S100B and myelin basic protein in the serum, have also been suggested as potential biomarkers of mTBI [82]. Two of the significantly up regulated miRNAs that were validated using the singleplex miRNA assay, have been shown to play a role in neuronal differentiation and CNS development. Normal miR-376a expression has been shown to be involved in the early fetal brain development, whereas accumulation of unedited miR-376a has been linked to neurodevelopmental disorders and increased metastasis potential of gliomas [83]–[86]. MiR-214 has also been shown to enhance neurite outgrowth and its expression is up regulated during the mouse cortical neuron, embryonic stem cells development, and at the early developmental stages in embryonic retina [87]–[88]. However, miR-214 levels have been shown to decrease in the neurons of the dorsal root ganglion after an injury to the sciatic nerve [89]. MiR-376a was also up regulated in the serum of rats exposed to blast induced moderate TBI [22]. Down regulated mir-106b and up regulated miR-376a and miR-214 along with other modulated miRNAs at the acute stage of injury, may reflect the metabolic active state of neural tissue engaged in the immediate response to injury that involve neuroprotection, synaptic plasticity, axonal damage/regeneration, and neurogenesis.

Endogenous neurogenesis and neuronal maturation have been proposed as keys to recovery and as therapeutic targets after TBI. TBI is often followed by the increase in endogenous neuronal progenitor cells proliferation and migration, neuronal differentiation, and neurogenesis [90]–[96]. Proliferating neuroprogenitor cells differentiate into immature neurons post TBI but fail to develop further into the mature neurons [91], [97]. A small subset of newborn granular neurons, however, has been observed in the brain after TBI, though the formation of these neurons is not significantly enhanced [97]–[98]. Dash and group also reported development of mature neurons in the dentate gyrus of the hippocampus one month after the TBI [96]. To determine if bioinformatics analysis targeting the axon guidance pathway may be exploited for predicting the severity of the injury, significantly modulated miRNA in each injury groups were analyzed by IPA for their predicted involvement in modulating axon guidance pathway. The number of miRNAs that are predicted to modulate axon guidance pathway related brain functions increased with the severity of the injury, suggesting an enhanced effect of injury on molecular functions associated with the axon guidance and neurogenesis (Figure 5). Long-term studies are needed to evaluate development of chronic pathologies such as axon damage, synapse and blood brain barrier functions in the brain following injury in this model of mTBI. However, the quantitative bioinformatics analysis of the modulated miRNAs relating to their potential CNS involvement can be helpful to identify the severity of the injury at early time points after the injury. In conclusion, a cohort of 13 serum miRNAs identified in this study, may be used as acute biomarkers of mTBI regardless of any neurobehavioral or cognitive alterations following the injury. Future studies will be needed to validate this cohort of 13 miRNAs as acute biomarkers of mTBI in the other animal models of injury and in the human mTBI. In addition, further behavioral studies will be needed to identify the long-term cognitive deficits and/or sleep disorders in the mTBI model described in this study. Such long-term studies will also be able to determine the prognostic value of the miRNAs identified in this study as acute biomarkers of mTBI.

## Supporting Information

Figure S1 **Closed head injury (CHI) device.** (A) CHI device was custom made based on the design of CHI device described by Flierl *et. al.* (2009) [30]. Injury was induced by a metal rod of specific weight falling under gravity. The rod can be set for a specific height using the holding grooves made 0.5 cm apart. The rod was released using a pneumatic control operated by a foot pedal. The tip of the rod was fitted with a rubber tip (1 mm in diameter/1 mm in length). (B) Site of the injury was over the parietal lobe, 2.5 mm from the sagittal and lamboid suture.
(TIF)

Figure S2 **Animal activity in an open field test.** Percentage of the animals that show reduced activity within each of the controls and the injury groups was evaluated. The lowest baseline horizontal and vertical activity values (beam breaks) in the naïve group were used as reference numbers. Animals exhibiting activity lower than the reference number were identified and were used to calculate the percentage of the animals within a group that exhibited reduced activity on day 1 post injury ([Number of animals that exhibited reduced activity in a group/total number of the animals in the group]*100). Data showed that as the grade of the injury increased, a higher percentage of the animals showed reduced activity.
(TIF)

Figure S3 **Motor activity post injury.** Animals were subjected to the rotarod test to evaluate the motor activity deficits post injury using a Med Associates rat rotarod (Med Associates, Inc., St. Albans, VT). Animals were placed on an accelerating (4–40 rpm) rotating rod (7.0 cm diameter) for a maximum period of 5 min and the time spent on the rod by each animal was measured. Three attempts of 5 min each were given to each animal and the mean time spent was calculated. Data presented here is the change scores between 1 day, 14 day and 30 day post injury and baseline (Mean time spent at each time point- mean time spent at BL measurement). No significant differences were found in the motor activity of the injured mice compared to the naïve or the sham groups at any time point measured.
(TIF)

Figure S4 **Hierarchical clustering (HC) of DDCt values.** HC of DDCt of miRNAs calculated over the sham controls showed 333 g injury groups (*i.e.*, IS3 and IS4) clustering together followed by IS2 and IS1 respectively. HC indicates that more severe of the injuries with in the mild spectrum showed close association.
(TIF)

Figure S5 **Brain functions related pathways targeted by the significantly modulated miRNAs common among all the four injury groups.** Thirteen common miRNAs that were significantly modulated among all the four injury groups were analyzed for their combined effect on KEGG pathways using DIANA-miRPath v2.0 software [50]. Eight of the significantly modulated miRNAs, miR-106b, miR-199a-3p, miR-214, miR-218, miR-31, miR-434-3p, miR-671-3p, and miR-574-3p were predicted to significantly modulate several nervous system function and disease related pathways.
(TIF)

Figure S6 **Brain functions related pathway targeted by the significantly modulated miRNAs unique to the injury groups with the neurobehavioral alterations.** Nineteen miRNAs that were significantly modulated among all the three injury groups, which demonstrated alteration in the neurobehavioral functions (*i.e.*, IS2, IS3 and IS4) were analyzed for their combined effect on the significant modulation of KEGG pathways using DIANA-miRPath v2.0 software [44].
(TIF)

Table S1 **List of the tasks performed for determining NSS-R scores.**
(DOCX)

Table S2 **NSS-R change scores.** NSS-R change scores of the individual groups are given and its significance with the other groups in the study is indicated. Values are expressed as mean ± SEM. * P value significant <0.05.
(DOCX)

Table S3 **The horizontal activity in OF.** The horizontal activity (number of beam breaks) of the individual groups is given and its significance with the other groups in the study is indicated. Values are expressed as mean ± SEM. * P value significant <0.05.
(DOCX)

Table S4 **The center time spent over the time period of the study.** The center time of the animals (in seconds) from all groups over the period of the study is given. Values are presented as mean ± SEM. * * P value significant <0.05.
(DOCX)

Table S5 **The center time of the animals in OFL.** The time spent in the center (in seconds) is given for each of the groups and its comparison with the other groups is given. Values are presented as mean ± SEM. * P value significant <0.05.
(DOCX)

Table S6 **The vertical activity over the time period of the study.** The vertical activity of the animals (number of beam breaks) from all the groups over the period of the study is given. Values are presented as mean ± SEM. * * P value significant <0.05.
(DOCX)

Table S7 **The vertical activity in OFL.** The vertical activity of the animals (number of beam breaks) in each group and its comparison with the other groups for day 1 is given. Values are presented as mean ± SEM. * P value significant <0.05.
(DOCX)

Table S8 **Day 1 ASR.** ASR response of the animals in each groups and its comparison with the other groups is given. Values are presented as mean ± SEM. * P value significant <0.05.
(DOCX)

Table S9 **Day 14 ASR.** ASR response of the animals in each groups and its comparison with the other groups is given. Values are presented as mean ± SEM. * P value significant <0.05.
(DOCX)

Table S10 **Average Ct values for the common miRNAs.** Average Ct value for each of the miRNA in the injury and the sham control group indicate their abundance in the sample. High expression  =  Ct ranging from 11–15; Good expression  =  Ct ranging from 16–20; Moderate expression  =  Ct ranging from 21–25; Low Expression  =  Ct ranging from 26–30; and Rare expression  =  Ct ranging from 31–35.
(DOCX)

Table S11 **MiRNAs present only in the injury groups that demonstrate behavior changes.** Nineteen miRNAs were significantly modulated in the injury group that showed significant behavior alterations. Of these 14 were up regulated and 5 were down regulated. Two miRNAs, mmu-miR-487b and mmu-miR-218-1* were expressed only in the injured animals and not in the sham-injured animals *i.e.*, “calibrator not detected”. For all other miRNAs Ct value was <36 in both, the injured and the sham-injured groups. Values are given as log10 of the fold change (2^−(mean ΔΔCt)^).
(DOCX)

Table S12 **MiRNA present only in the injury groups that do not demonstrate behavior changes.** Two miRNAs, miR-297a* and miR-181c were found to be significantly modulated in the IS1 injury group, which did not demonstrate any significant neurobehavioral and cognitive changes as compared to the sham injury group. “Calibrator not detected” is the miRNA which is expressed only in the injured animals and not in the un-injured sham animals. “Target not detected” is the miRNA that was expressed only in the un-injured sham animals and not in the injured animals. In such case the Ct value for non-detected miRNA was taken as 40 followed by the fold change and statistical analysis.
(DOCX)

Table S13 **Selection of the endogenous control miRNA.** The most stable miRNA were selected based on their Ct values across the samples (n = 30). Ct for each miRNA from all the samples, irrespective of the injury or control group, were taken and Ct values with a SD <1 were selected. Mean Ct were calculated to determine the abundance of miRNAs in the serum samples. To ensure that there are no outliers, median Ct was also calculated. MiRNA that showed similar mean and median Ct values were then selected. MiRNA that show more abundance *i.e.*, Ct <25 were considered for determining a candidate stable endogenous miRNA in validation experiments.
(DOCX)

## References

[1] Risdall JE, Menon DK (2011) Traumatic brain injury. Philosophical transactions of the Royal Society of London Series B, Biological sciences 366: 241–250. 21149359 10.1098/rstb.2010.0230 PMC3013429

[2] Teasdale G, Jennett B (1976) Assessment and prognosis of coma after head injury. Acta neurochirurgica 34: 45–55. 961490 10.1007/BF01405862

[3] Fischer H (2013) U.S. Military Casualty Statistics: Operation New Dawn, Operation Iraqi Freedom, and Operation Enduring Freedom. CRS Report for Congress. Prepared for Members and Committees of Congress: Congressional Research Service. pp. 1–12.

[4] Belanger HG, Vanderploeg RD, Curtiss G, Warden DL (2007) Recent neuroimaging techniques in mild traumatic brain injury. The Journal of neuropsychiatry and clinical neurosciences 19: 5–20. 17308222 10.1176/jnp.2007.19.1.5

[5] Niogi SN, Mukherjee P, Ghajar J, Johnson C, Kolster RA, et al (2008) Extent of microstructural white matter injury in postconcussive syndrome correlates with impaired cognitive reaction time: a 3T diffusion tensor imaging study of mild traumatic brain injury. AJNR American journal of neuroradiology 29: 967–973. 18272556 10.3174/ajnr.A0970 PMC8128563

[6] Yuh EL, Cooper SR, Ferguson AR, Manley GT (2012) Quantitative CT improves outcome prediction in acute traumatic brain injury. Journal of neurotrauma 29: 735–746. 21970562 10.1089/neu.2011.2008 PMC3303103

[7] Vos PE, Jacobs B, Andriessen TM, Lamers KJ, Borm GF, et al (2010) GFAP and S100B are biomarkers of traumatic brain injury: an observational cohort study. Neurology 75: 1786–1793. 21079180 10.1212/WNL.0b013e3181fd62d2

[8] Thelin EP, Johannesson L, Nelson D, Bellander BM (2013) S100B Is an Important Outcome Predictor in Traumatic Brain Injury. Journal of neurotrauma 30: 519–528. 23297751 10.1089/neu.2012.2553

[9] Papa L, Lewis LM, Falk JL, Zhang Z, Silvestri S, et al (2012) Elevated levels of serum glial fibrillary acidic protein breakdown products in mild and moderate traumatic brain injury are associated with intracranial lesions and neurosurgical intervention. Annals of emergency medicine 59: 471–483. 22071014 10.1016/j.annemergmed.2011.08.021 PMC3830977

[10] Papa L, Lewis LM, Silvestri S, Falk JL, Giordano P, et al (2012) Serum levels of ubiquitin C-terminal hydrolase distinguish mild traumatic brain injury from trauma controls and are elevated in mild and moderate traumatic brain injury patients with intracranial lesions and neurosurgical intervention. The journal of trauma and acute care surgery 72: 1335–1344. 22673263 10.1097/TA.0b013e3182491e3d PMC5516044

[11] Papa L, Akinyi L, Liu MC, Pineda JA, Tepas JJ 3rd, et al (2010) Ubiquitin C-terminal hydrolase is a novel biomarker in humans for severe traumatic brain injury. Critical care medicine 38: 138–144. 19726976 10.1097/CCM.0b013e3181b788ab PMC3445330

[12] Brophy GM, Mondello S, Papa L, Robicsek SA, Gabrielli A, et al (2011) Biokinetic analysis of ubiquitin C-terminal hydrolase-L1 (UCH-L1) in severe traumatic brain injury patient biofluids. Journal of neurotrauma 28: 861–870. 21309726 10.1089/neu.2010.1564 PMC3113451

[13] Pogoda TK, Hendricks AM, Iverson KM, Stolzmann KL, Krengel MH, et al (2012) Multisensory impairment reported by veterans with and without mild traumatic brain injury history. Journal of rehabilitation research and development 49: 971–984. 23341273 10.1682/jrrd.2011.06.0099

[14] Theeler BJ, Flynn FG, Erickson JC (2012) Chronic daily headache in U.S. soldiers after concussion. Headache 52: 732–738. 22404747 10.1111/j.1526-4610.2012.02112.x

[15] Kiraly M, Kiraly SJ (2007) Traumatic brain injury and delayed sequelae: a review–traumatic brain injury and mild traumatic brain injury (concussion) are precursors to later-onset brain disorders, including early-onset dementia. TheScientificWorldJournal 7: 1768–1776. 10.1100/tsw.2007.269 PMC5901335 18040539

[16] Mayr M, Zampetaki A, Willeit P, Willeit J, Kiechl S (2013) MicroRNAs within the continuum of postgenomics biomarker discovery. Arteriosclerosis, thrombosis, and vascular biology 33: 206–214. 10.1161/ATVBAHA.112.300141 23325478

[17] Allegra A, Alonci A, Campo S, Penna G, Petrungaro A, et al (2012) Circulating microRNAs: new biomarkers in diagnosis, prognosis and treatment of cancer (review). International journal of oncology 41: 1897–1912. 23026890 10.3892/ijo.2012.1647

[18] Wahid F, Shehzad A, Khan T, Kim YY (2010) MicroRNAs: synthesis, mechanism, function, and recent clinical trials. Biochimica et biophysica acta 1803: 1231–1243. 20619301 10.1016/j.bbamcr.2010.06.013

[19] Redell JB, Liu Y, Dash PK (2009) Traumatic brain injury alters expression of hippocampal microRNAs: potential regulators of multiple pathophysiological processes. Journal of neuroscience research 87: 1435–1448. 19021292 10.1002/jnr.21945 PMC5980641

[20] Redell JB, Zhao J, Dash PK (2011) Altered expression of miRNA-21 and its targets in the hippocampus after traumatic brain injury. Journal of neuroscience research 89: 212–221. 21162128 10.1002/jnr.22539 PMC7958494

[21] Lei P, Li Y, Chen X, Yang S, Zhang J (2009) Microarray based analysis of microRNA expression in rat cerebral cortex after traumatic brain injury. Brain research 1284: 191–201. 19501075 10.1016/j.brainres.2009.05.074

[22] Balakathiresan N, Bhomia M, Chandran R, Chavko M, McCarron RM, et al (2012) MicroRNA let-7i is a promising serum biomarker for blast-induced traumatic brain injury. Journal of neurotrauma 29: 1379–1387. 22352906 10.1089/neu.2011.2146 PMC3335133

[23] Truettner JS, Alonso OF, Bramlett HM, Dietrich WD (2011) Therapeutic hypothermia alters microRNA responses to traumatic brain injury in rats. Journal of cerebral blood flow and metabolism: official journal of the International Society of Cerebral Blood Flow and Metabolism 31: 1897–1907. 10.1038/jcbfm.2011.33 PMC3185878 21505482

[24] Redell JB, Moore AN, Ward NH 3rd, Hergenroeder GW, Dash PK (2010) Human traumatic brain injury alters plasma microRNA levels. Journal of neurotrauma 27: 2147–2156. 20883153 10.1089/neu.2010.1481 PMC6468948

[25] Flierl MA, Stahel PF, Beauchamp KM, Morgan SJ, Smith WR, et al (2009) Mouse closed head injury model induced by a weight-drop device. Nature protocols 4: 1328–1337. 19713954 10.1038/nprot.2009.148

[26] Grunberg NE, Yarnell AM, Hamilton KR, Starosciak AK, Chwa A, et al.. (2011) A revised neurological severity scale for rodents.. Annual Meeting of Society for Neuroscience. Washington, D.C.

[27] Sharma P, Su YA, Barry ES, Grunberg NE, Lei Z (2012) Mitochondrial targeted neuron focused genes in hippocampus of rats with traumatic brain injury. International journal of critical illness and injury science 2: 172–179. 23181213 10.4103/2229-5151.100931 PMC3500011

[28] Shohami E, Novikov M, Bass R (1995) Long-term effect of HU-211, a novel non-competitive NMDA antagonist, on motor and memory functions after closed head injury in the rat. Brain research 674: 55–62. 7773695 10.1016/0006-8993(94)01433-i

[29] Hamm RJ (2001) Neurobehavioral assessment of outcome following traumatic brain injury in rats: an evaluation of selected measures. Journal of neurotrauma 18: 1207–1216. 11721739 10.1089/089771501317095241

[30] Marti M, Mela F, Fantin M, Zucchini S, Brown JM, et al (2005) Blockade of nociceptin/orphanin FQ transmission attenuates symptoms and neurodegeneration associated with Parkinson's disease. The Journal of neuroscience: the official journal of the Society for Neuroscience 25: 9591–9601. 16237164 10.1523/JNEUROSCI.2546-05.2005 PMC6725738

[31] Yarnell AM, Shaughness MC, Barry ES, Ahlers ST, McCarron RM, et al (2013) Blast traumatic brain injury in the rat using a blast overpressure model. Current protocols in neuroscience/editorial board Jacqueline N Crawley [et al] Chapter 9 Unit 9 41. 10.1002/0471142301.ns0941s62 23315947

[32] Xing G, Barry ES, Benford B, Grunberg NE, Li H, et al (2013) Impact of repeated stress on traumatic brain injury-induced mitochondrial electron transport chain expression and behavioral responses in rats. Frontiers in neurology 4: 196. 24376434 10.3389/fneur.2013.00196 PMC3859919

[33] Bowen DJ, Eury SE, Grunberg NE (1986) Nicotine's effects on female rats' body weight: caloric intake and physical activity. Pharmacology, biochemistry, and behavior 25: 1131–1136. 10.1016/0091-3057(86)90099-7 3809215

[34] Elliott BM, Faraday MM, Phillips JM, Grunberg NE (2004) Effects of nicotine on elevated plus maze and locomotor activity in male and female adolescent and adult rats. Pharmacology, biochemistry, and behavior 77: 21–28. 10.1016/j.pbb.2003.09.016 14724038

[35] Faraday MM, O'Donoghue VA, Grunberg NE (2003) Effects of nicotine and stress on locomotion in Sprague-Dawley and Long-Evans male and female rats. Pharmacology, biochemistry, and behavior 74: 325–333. 10.1016/s0091-3057(02)00999-1 12479951

[36] Grunberg NE, Bowen DJ (1985) The role of physical activity in nicotine's effects on body weight. Pharmacology, biochemistry, and behavior 23: 851–854. 10.1016/0091-3057(85)90081-4 4080769

[37] Morse DE, Davis HD, Popke EJ, Brown KJ, O'Donoghue VA, et al (1997) Effects of ddC and AZT on locomotion and acoustic startle. I: Acute effects in female rats. Pharmacology, biochemistry, and behavior 56: 221–228. 10.1016/s0091-3057(96)00214-6 9050078

[38] Hamilton KR, Starosciak AK, Chwa A, Grunberg NE (2012) Nicotine behavioral sensitization in Lewis and Fischer male rats. Experimental and clinical psychopharmacology 20: 345–351. 22775443 10.1037/a0029088

[39] Acri JB, Grunberg NE, Morse DE (1991) Effects of nicotine on the acoustic startle reflex amplitude in rats. Psychopharmacology 104: 244–248. 1876669 10.1007/BF02244186

[40] Alain C, He Y, Grady C (2008) The contribution of the inferior parietal lobe to auditory spatial working memory. Journal of cognitive neuroscience 20: 285–295. 18275335 10.1162/jocn.2008.20014

[41] Swerdlow NR, Caine SB, Braff DL, Geyer MA (1992) The neural substrates of sensorimotor gating of the startle reflex: a review of recent findings and their implications. Journal of psychopharmacology (Oxford, England) 6: 176–190. 10.1177/026988119200600210 22291349

[42] Acri JB, Morse DE, Popke EJ, Grunberg NE (1994) Nicotine increases sensory gating measured as inhibition of the acoustic startle reflex in rats. Psychopharmacology 114: 369–374. 7838931 10.1007/BF02244861

[43] Faraday MM, Rahman MA, Scheufele PM, Grunberg NE (1998) Nicotine administration impairs sensory gating in Long-Evans rats. Pharmacology, biochemistry, and behavior 61: 281–289. 10.1016/s0091-3057(98)00094-x 9768562

[44] Allison PD (1990) Change Scores as Dependent Variables in Regression Analysis. Sociological Methodology 20: 93–114.

[45] D'Haene B, Mestdagh P, Hellemans J, Vandesompele J (2012) miRNA expression profiling: from reference genes to global mean normalization. Methods in molecular biology (Clifton, NJ) 822: 261–272. 10.1007/978-1-61779-427-8_18 22144205

[46] Deo A, Carlsson J, Lindlof A (2011) How to choose a normalization strategy for miRNA quantitative real-time (qPCR) arrays. Journal of bioinformatics and computational biology 9: 795–812. 22084014 10.1142/s0219720011005793

[47] Marmarou A, Foda MA, van den Brink W, Campbell J, Kita H, et al (1994) A new model of diffuse brain injury in rats. Part I: Pathophysiology and biomechanics. Journal of neurosurgery 80: 291–300. 8283269 10.3171/jns.1994.80.2.0291

[48] Foda MA, Marmarou A (1994) A new model of diffuse brain injury in rats. Part II: Morphological characterization. Journal of neurosurgery 80: 301–313. 8283270 10.3171/jns.1994.80.2.0301

[49] Vlachos IS, Kostoulas N, Vergoulis T, Georgakilas G, Reczko M, et al (2012) DIANA miRPath v.2.0: investigating the combinatorial effect of microRNAs in pathways. Nucleic acids research 40: W498–504. 22649059 10.1093/nar/gks494 PMC3394305

[50] Song J, Bai Z, Han W, Zhang J, Meng H, et al (2012) Identification of suitable reference genes for qPCR analysis of serum microRNA in gastric cancer patients. Digestive diseases and sciences 57: 897–904. 22198701 10.1007/s10620-011-1981-7

[51] Rapp PE, Curley KC (2012) Is a diagnosis of “mild traumatic brain injury” a category mistake? The journal of trauma and acute care surgery 73: S13–23. 22847083 10.1097/TA.0b013e318260604b

[52] Bryan CJ, Clemans TA, Hernandez AM, Rudd MD (2013) Loss of consciousness, depression, posttraumatic stress disorder, and suicide risk among deployed military personnel with mild traumatic brain injury. The Journal of head trauma rehabilitation 28: 13–20. 23076097 10.1097/HTR.0b013e31826c73cc

[53] Katz RJ, Roth KA, Carroll BJ (1981) Acute and chronic stress effects on open field activity in the rat: implications for a model of depression. Neuroscience and biobehavioral reviews 5: 247–251. 7196554 10.1016/0149-7634(81)90005-1

[54] Seligman ME, Maier SF (1967) Failure to escape traumatic shock. Journal of experimental psychology 74: 1–9. 6032570 10.1037/h0024514

[55] Overmier JB, Seligman ME (1967) Effects of inescapable shock upon subsequent escape and avoidance responding. Journal of comparative and physiological psychology 63: 28–33. 6029715 10.1037/h0024166

[56] Kumar S, Rao SL, Nair RG, Pillai S, Chandramouli BA, et al (2005) Sensory gating impairment in development of post-concussive symptoms in mild head injury. Psychiatry and clinical neurosciences 59: 466–472. 16048453 10.1111/j.1440-1819.2005.01400.x

[57] Lee Y, Lopez DE, Meloni EG, Davis M (1996) A primary acoustic startle pathway: obligatory role of cochlear root neurons and the nucleus reticularis pontis caudalis. The Journal of neuroscience: the official journal of the Society for Neuroscience 16: 3775–3789. 8642420 10.1523/JNEUROSCI.16-11-03775.1996 PMC6578836

[58] Koch M, Schnitzler HU (1997) The acoustic startle response in rats–circuits mediating evocation, inhibition and potentiation. Behavioural brain research 89: 35–49. 9475613 10.1016/s0166-4328(97)02296-1

[59] Freund HJ (2003) Somatosensory and motor disturbances in patients with parietal lobe lesions. Advances in neurology 93: 179–193. 12894408

[60] Leung AW, Alain C (2011) Working memory load modulates the auditory “What” and “Where” neural networks. NeuroImage 55: 1260–1269. 21195187 10.1016/j.neuroimage.2010.12.055

[61] Liu L, Sun T, Liu Z, Chen X, Zhao L, et al (2014) Traumatic Brain Injury Dysregulates MicroRNAs to Modulate Cell Signaling in Rat Hippocampus. PloS one 9: e103948. 25089700 10.1371/journal.pone.0103948 PMC4121204

[62] Sabirzhanov B, Zhao Z, Stoica BA, Loane DJ, Wu J, et al (2014) Downregulation of miR-23a and miR-27a following Experimental Traumatic Brain Injury Induces Neuronal Cell Death through Activation of Proapoptotic Bcl-2 Proteins. The Journal of neuroscience: the official journal of the Society for Neuroscience 34: 10055–10071. 25057207 10.1523/JNEUROSCI.1260-14.2014 PMC4107397

[63] Bao TH, Miao W, Han JH, Yin M, Yan Y, et al.. (2014) Spontaneous Running Wheel Improves Cognitive Functions of Mouse Associated with miRNA Expressional Alteration in Hippocampus Following Traumatic Brain Injury. Journal of molecular neuroscience: MN. 10.1007/s12031-014-0344-1 24920273

[64] Moon C, Ahn M, Kim S, Jin JK, Sim KB, et al (2004) Temporal patterns of the embryonic intermediate filaments nestin and vimentin expression in the cerebral cortex of adult rats after cryoinjury. Brain research 1028: 238–242. 15527750 10.1016/j.brainres.2004.09.022

[65] Hausmann R, Betz P (2001) Course of glial immunoreactivity for vimentin, tenascin and alpha1-antichymotrypsin after traumatic injury to human brain. International journal of legal medicine 114: 338–342. 11508799 10.1007/s004140000199

[66] Wang G, Jiang X, Pu H, Zhang W, An C, et al (2013) Scriptaid, a novel histone deacetylase inhibitor, protects against traumatic brain injury via modulation of PTEN and AKT pathway: scriptaid protects against TBI via AKT. Neurotherapeutics: the journal of the American Society for Experimental NeuroTherapeutics 10: 124–142. 23132328 10.1007/s13311-012-0157-2 PMC3557358

[67] Goh CP, Putz U, Howitt J, Low LH, Gunnersen J, et al (2014) Nuclear trafficking of Pten after brain injury leads to neuron survival not death. Experimental neurology 252: 37–46. 24275527 10.1016/j.expneurol.2013.11.017

[68] Yang K, Perez-Polo JR, Mu XS, Yan HQ, Xue JJ, et al (1996) Increased expression of brain-derived neurotrophic factor but not neurotrophin-3 mRNA in rat brain after cortical impact injury. Journal of neuroscience research 44: 157–164. 8723224 10.1002/(SICI)1097-4547(19960415)44:2<157::AID-JNR8>3.0.CO;2-C

[69] Van Den Heuvel C, Lewis S, Wong M, Manavis J, Finnie J, et al.. (1998) Diffuse neuronal perikaryon amyloid precursor protein immunoreactivity in a focal head impact model. Acta neurochirurgica Supplement 71: 209–211. 10.1007/978-3-7091-6475-4_60 9779186

[70] Griesbach GS, Hovda DA, Molteni R, Gomez-Pinilla F (2002) Alterations in BDNF and synapsin I within the occipital cortex and hippocampus after mild traumatic brain injury in the developing rat: reflections of injury-induced neuroplasticity. Journal of neurotrauma 19: 803–814. 12184851 10.1089/08977150260190401

[71] Itoh T, Satou T, Nishida S, Tsubaki M, Hashimoto S, et al (2009) Improvement of cerebral function by anti-amyloid precursor protein antibody infusion after traumatic brain injury in rats. Molecular and cellular biochemistry 324: 191–199. 19130181 10.1007/s11010-008-0013-1

[72] Murakami N, Yamaki T, Iwamoto Y, Sakakibara T, Kobori N, et al (1998) Experimental brain injury induces expression of amyloid precursor protein, which may be related to neuronal loss in the hippocampus. Journal of neurotrauma 15: 993–1003. 9840772 10.1089/neu.1998.15.993

[73] Corrigan F, Thornton E, Roisman LC, Leonard AV, Vink R, et al (2014) The neuroprotective activity of the amyloid precursor protein against traumatic brain injury is mediated via the heparin binding site in residues 96-110. Journal of neurochemistry 128: 196–204. 23919582 10.1111/jnc.12391

[74] Corrigan F, Vink R, Blumbergs PC, Masters CL, Cappai R, et al (2012) Characterisation of the effect of knockout of the amyloid precursor protein on outcome following mild traumatic brain injury. Brain research 1451: 87–99. 22424792 10.1016/j.brainres.2012.02.045

[75] Hanell A, Clausen F, Djupsjo A, Vallstedt A, Patra K, et al (2012) Functional and histological outcome after focal traumatic brain injury is not improved in conditional EphA4 knockout mice. Journal of neurotrauma 29: 2660–2671. 22985250 10.1089/neu.2012.2376

[76] Takeuchi S, Nawashiro H, Sato S, Kawauchi S, Nagatani K, et al (2013) A better mild traumatic brain injury model in the rat. Acta neurochirurgica Supplement 11899–101. 10.1007/978-3-7091-1434-6_17 23564112

[77] Selwyn R, Hockenbury N, Jaiswal S, Mathur S, Armstrong RC, et al (2013) Mild traumatic brain injury results in depressed cerebral glucose uptake: An (18)FDG PET study. Journal of neurotrauma 30: 1943–1953. 23829400 10.1089/neu.2013.2928

[78] Johnson VE, Stewart W, Smith DH (2013) Axonal pathology in traumatic brain injury. Experimental neurology 246: 35–43. 22285252 10.1016/j.expneurol.2012.01.013 PMC3979341

[79] Schirmer L, Merkler D, Konig FB, Bruck W, Stadelmann C (2013) Neuroaxonal regeneration is more pronounced in early multiple sclerosis than in traumatic brain injury lesions. Brain pathology (Zurich, Switzerland) 23: 2–12. 10.1111/j.1750-3639.2012.00608.x PMC8057635 22612622

[80] Urrea C, Castellanos DA, Sagen J, Tsoulfas P, Bramlett HM, et al (2007) Widespread cellular proliferation and focal neurogenesis after traumatic brain injury in the rat. Restorative neurology and neuroscience 25: 65–76. 17473396

[81] Frugier T, Conquest A, McLean C, Currie P, Moses D, et al (2012) Expression and activation of EphA4 in the human brain after traumatic injury. Journal of neuropathology and experimental neurology 71: 242–250. 22318127 10.1097/NEN.0b013e3182496149

[82] Rostami E, Davidsson J, Ng KC, Lu J, Gyorgy A, et al (2012) A Model for Mild Traumatic Brain Injury that Induces Limited Transient Memory Impairment and Increased Levels of Axon Related Serum Biomarkers. Frontiers in neurology 3: 115. 22837752 10.3389/fneur.2012.00115 PMC3401945

[83] Goossens K, Mestdagh P, Lefever S, Van Poucke M, Van Zeveren A, et al.. (2013) Regulatory microRNA network identification in bovine blastocyst development. Stem cells and development. 10.1089/scd.2012.0708 PMC3685315 23398486

[84] Kawahara Y, Zinshteyn B, Sethupathy P, Iizasa H, Hatzigeorgiou AG, et al (2007) Redirection of silencing targets by adenosine-to-inosine editing of miRNAs. Science (New York, NY) 315: 1137–1140. 10.1126/science.1138050 PMC2953418 17322061

[85] Ekdahl Y, Farahani HS, Behm M, Lagergren J, Ohman M (2012) A-to-I editing of microRNAs in the mammalian brain increases during development. Genome research 22: 1477–1487. 22645261 10.1101/gr.131912.111 PMC3409261

[86] Choudhury Y, Tay FC, Lam DH, Sandanaraj E, Tang C, et al (2012) Attenuated adenosine-to-inosine editing of microRNA-376a* promotes invasiveness of glioblastoma cells. The Journal of clinical investigation 122: 4059–4076. 23093778 10.1172/JCI62925 PMC3484441

[87] Chen H, Shalom-Feuerstein R, Riley J, Zhang SD, Tucci P, et al (2010) miR-7 and miR-214 are specifically expressed during neuroblastoma differentiation, cortical development and embryonic stem cells differentiation, and control neurite outgrowth in vitro. Biochemical and biophysical research communications 394: 921–927. 20230785 10.1016/j.bbrc.2010.03.076

[88] Decembrini S, Bressan D, Vignali R, Pitto L, Mariotti S, et al (2009) MicroRNAs couple cell fate and developmental timing in retina. Proceedings of the National Academy of Sciences of the United States of America 106: 21179–21184. 19965369 10.1073/pnas.0909167106 PMC2781736

[89] Zhang HY, Zheng SJ, Zhao JH, Zhao W, Zheng LF, et al (2011) MicroRNAs 144, 145, and 214 are down-regulated in primary neurons responding to sciatic nerve transection. Brain research 1383: 62–70. 21276775 10.1016/j.brainres.2011.01.067

[90] Rice AC, Khaldi A, Harvey HB, Salman NJ, White F, et al (2003) Proliferation and neuronal differentiation of mitotically active cells following traumatic brain injury. Experimental neurology 183: 406–417. 14552881 10.1016/s0014-4886(03)00241-3

[91] Yi X, Jin G, Zhang X, Mao W, Li H, et al (2013) Cortical endogenic neural regeneration of adult rat after traumatic brain injury. PloS one 8: e70306. 23922973 10.1371/journal.pone.0070306 PMC3726380

[92] Zhang L, Yan R, Zhang Q, Wang H, Kang X, et al (2013) Survivin, a key component of the Wnt/beta-catenin signaling pathway, contributes to traumatic brain injury-induced adult neurogenesis in the mouse dentate gyrus. International journal of molecular medicine 32: 867–875. 23900556 10.3892/ijmm.2013.1456

[93] Itoh T, Satou T, Hashimoto S, Ito H (2005) Isolation of neural stem cells from damaged rat cerebral cortex after traumatic brain injury. Neuroreport 16: 1687–1691. 16189478 10.1097/01.wnr.0000183330.44112.ab

[94] Zheng W, ZhuGe Q, Zhong M, Chen G, Shao B, et al (2013) Neurogenesis in adult human brain after traumatic brain injury. Journal of neurotrauma 30: 1872–1880. 21275797 10.1089/neu.2010.1579 PMC3815038

[95] Rola R, Mizumatsu S, Otsuka S, Morhardt DR, Noble-Haeusslein LJ, et al (2006) Alterations in hippocampal neurogenesis following traumatic brain injury in mice. Experimental neurology 202: 189–199. 16876159 10.1016/j.expneurol.2006.05.034

[96] Dash PK, Mach SA, Moore AN (2001) Enhanced neurogenesis in the rodent hippocampus following traumatic brain injury. Journal of neuroscience research 63: 313–319. 11170181 10.1002/1097-4547(20010215)63:4<313::AID-JNR1025>3.0.CO;2-4

[97] Gao X, Chen J (2013) Moderate traumatic brain injury promotes neural precursor proliferation without increasing neurogenesis in the adult hippocampus. Experimental neurology 239: 38–48. 23022454 10.1016/j.expneurol.2012.09.012 PMC3755608

[98] Bye N, Carron S, Han X, Agyapomaa D, Ng SY, et al (2011) Neurogenesis and glial proliferation are stimulated following diffuse traumatic brain injury in adult rats. Journal of neuroscience research 89: 986–1000. 21488090 10.1002/jnr.22635

