# Supplementary figures and images for: Identification of Serum MicroRNA Signatures for Diagnosis of Mild Traumatic Brain Injury in a Closed Head Injury Model
Source: PLoS One. 2014 Nov 7;9(11):e112019. doi: 10.1371/journal.pone.0112019 (PMC4224512; doi:10.1371/journal.pone.0112019)

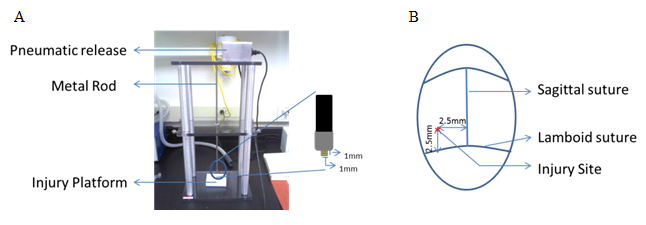

Supplement: Figure S1 — Closed head injury (CHI) device. (A) CHI device was custom made based on the design of CHI device described by Flierl et. al. (2009) [30]. Injury was induced by a metal rod of specific weight falling under gravity. The rod can be set for a specific height using the holding grooves made 0.5 cm apart. The rod was released using a pneumatic control operated by a foot pedal. The tip of the rod was fitted with a rubber tip (1 mm in diameter/1 mm in length). (B) Site of the injury was over the parietal lobe, 2.5 mm from the sagittal and lamboid suture. (TIF) [file pone.0112019.s001.tif]

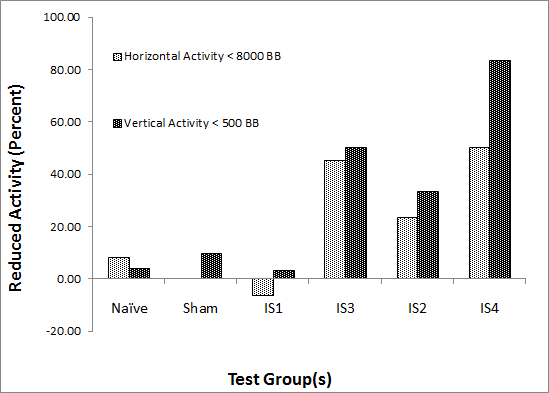

Supplement: Figure S2 — Animal activity in an open field test. Percentage of the animals that show reduced activity within each of the controls and the injury groups was evaluated. The lowest baseline horizontal and vertical activity values (beam breaks) in the naïve group were used as reference numbers. Animals exhibiting activity lower than the reference number were identified and were used to calculate the percentage of the animals within a group that exhibited reduced activity on day 1 post injury ([Number of animals that exhibited reduced activity in a group/total number of the animals in the group]*100). Data showed that as the grade of the injury increased, a higher percentage of the animals showed reduced activity. (TIF) [file pone.0112019.s002.tif]

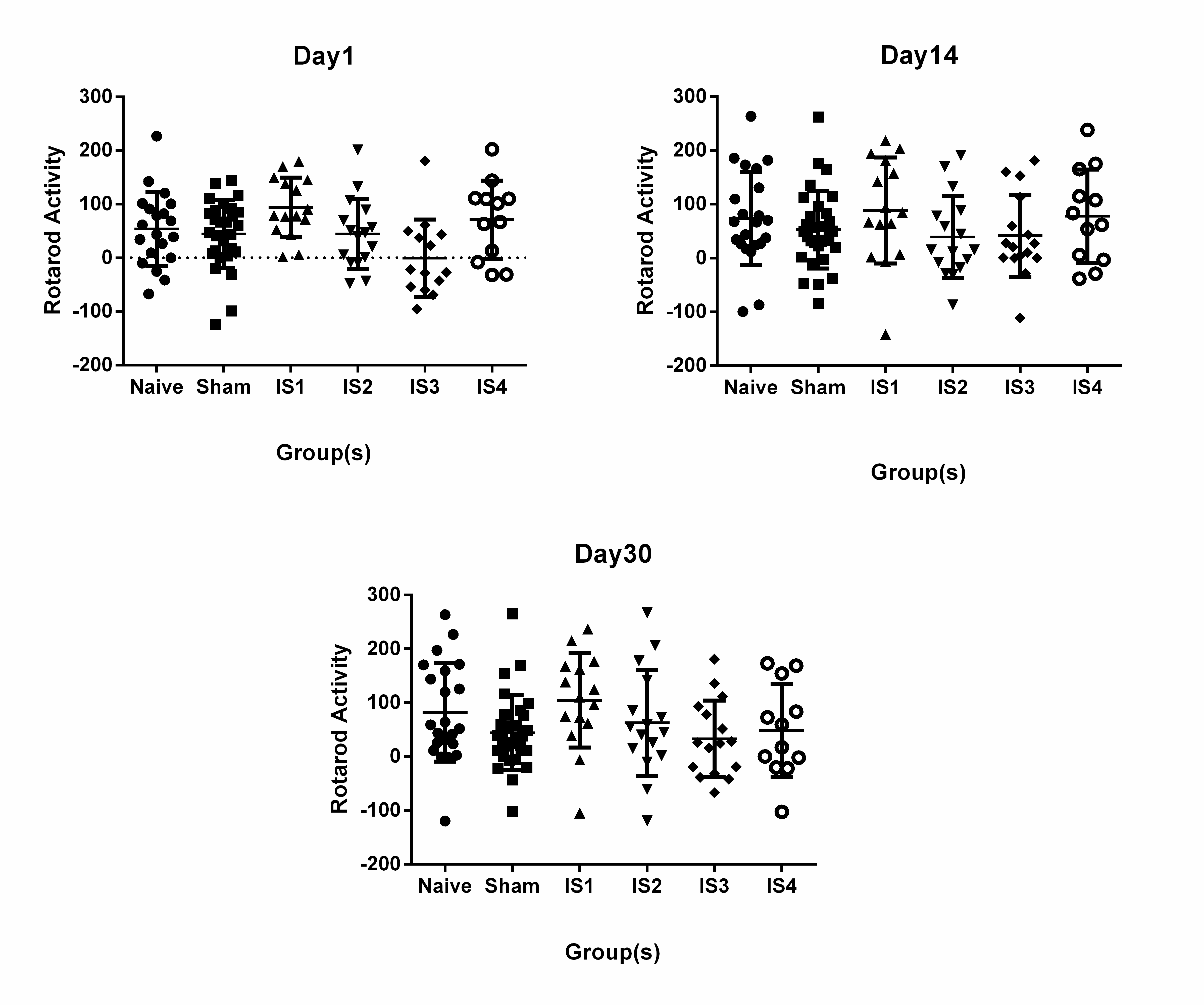

Supplement: Figure S3 — Motor activity post injury. Animals were subjected to the rotarod test to evaluate the motor activity deficits post injury using a Med Associates rat rotarod (Med Associates, Inc., St. Albans, VT). Animals were placed on an accelerating (4–40 rpm) rotating rod (7.0 cm diameter) for a maximum period of 5 min and the time spent on the rod by each animal was measured. Three attempts of 5 min each were given to each animal and the mean time spent was calculated. Data presented here is the change scores between 1 day, 14 day and 30 day post injury and baseline (Mean time spent at each time point- mean time spent at BL measurement). No significant differences were found in the motor activity of the injured mice compared to the naïve or the sham groups at any time point measured. (TIF) [file pone.0112019.s003.tif]

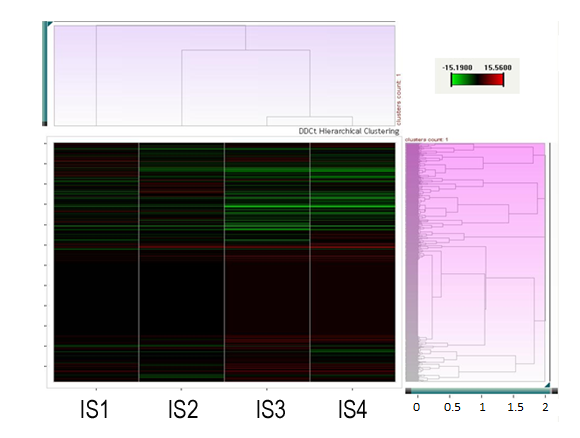

Supplement: Figure S4 — Hierarchical clustering (HC) of DDCt values. HC of DDCt of miRNAs calculated over the sham controls showed 333 g injury groups (i.e., IS3 and IS4) clustering together followed by IS2 and IS1 respectively. HC indicates that more severe of the injuries with in the mild spectrum showed close association. (TIF) [file pone.0112019.s004.tif]

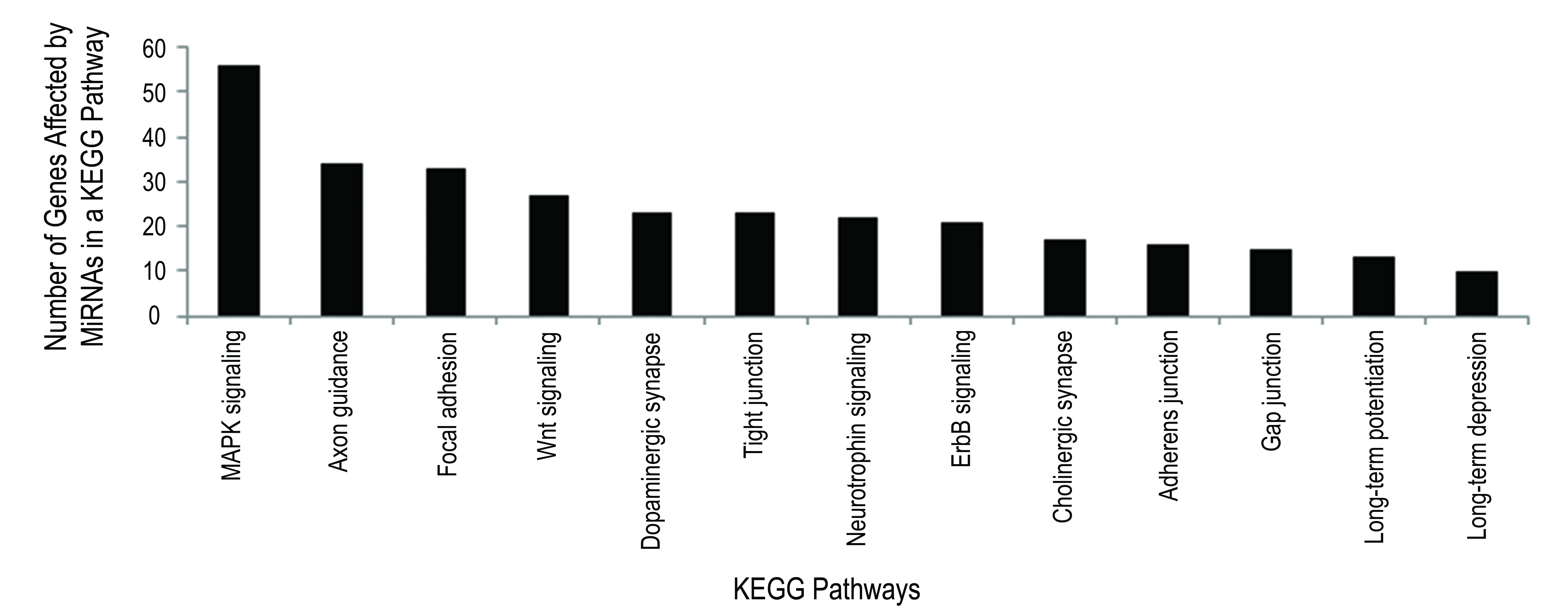

Supplement: Figure S5 — Brain functions related pathways targeted by the significantly modulated miRNAs common among all the four injury groups. Thirteen common miRNAs that were significantly modulated among all the four injury groups were analyzed for their combined effect on KEGG pathways using DIANA-miRPath v2.0 software [50]. Eight of the significantly modulated miRNAs, miR-106b, miR-199a-3p, miR-214, miR-218, miR-31, miR-434-3p, miR-671-3p, and miR-574-3p were predicted to significantly modulate several nervous system function and disease related pathways. (TIF) [file pone.0112019.s005.tif]

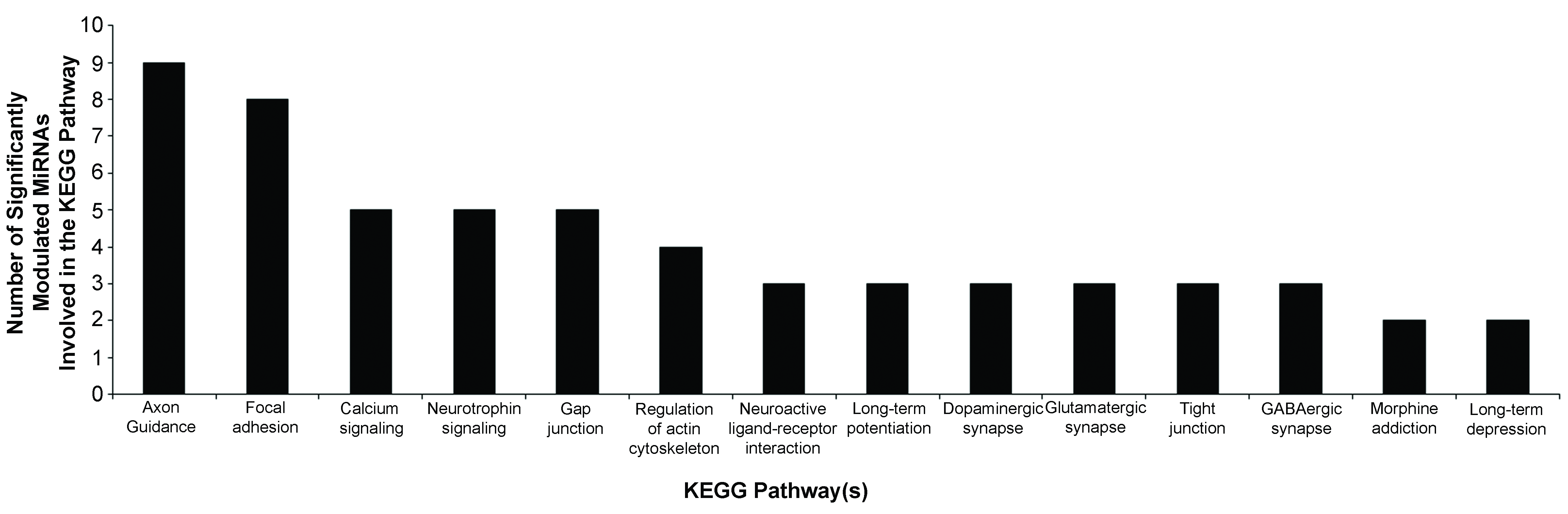

Supplement: Figure S6 — Brain functions related pathway targeted by the significantly modulated miRNAs unique to the injury groups with the neurobehavioral alterations. Nineteen miRNAs that were significantly modulated among all the three injury groups, which demonstrated alteration in the neurobehavioral functions (i.e., IS2, IS3 and IS4) were analyzed for their combined effect on the significant modulation of KEGG pathways using DIANA-miRPath v2.0 software [44]. (TIF) [file pone.0112019.s006.tif]
