# Supplementary material for: Identification of Serum MicroRNA Signatures for Diagnosis of Mild Traumatic Brain Injury in a Closed Head Injury Model
Source: PLoS One. 2014 Nov 7;9(11):e112019. doi: 10.1371/journal.pone.0112019 (PMC4224512; doi:10.1371/journal.pone.0112019)
Supplement: Table S1 — List of the tasks performed for determining NSS-R scores. (DOCX) [file pone.0112019.s007.docx]

**Table S1**: List of the tasks performed for determining NSS-R scores.

| Activity Test | Score | | |
| --- | --- | --- | --- |
|  | 0 | 1 | 2 |
| General balance | Balance and walk | Balance/no walk | No balance/fall |
| Landing | Normal reflex | Partial reflex/unbalanced | No reflex/falls flat |
| Tail raise | Normal Reflex | Partial reflex/weak | No reflex/limp |
| Drag | Walking motion | Partial/unilateral | No response/drag |
| Righting reflex | Instant | Delayed or with effort | No response |
| Ear reflex | Full response | Partial response | No response |
| Eye reflex | Blink | Partial response | No response |
| Sound reflex | Flinch and walk | Flinch and pause/ Partial Jump/ Walk ignore | Startle and freeze/ Strong jump |
| Foot reflex | Turn and bite | Turn/no bite | No response |
| Tail reflex | Turn and bite | Turn/no bite | No response |
