# Supplementary material for: Identification of Serum MicroRNA Signatures for Diagnosis of Mild Traumatic Brain Injury in a Closed Head Injury Model
Source: PLoS One. 2014 Nov 7;9(11):e112019. doi: 10.1371/journal.pone.0112019 (PMC4224512; doi:10.1371/journal.pone.0112019)
Supplement: Table S2 — NSS-R change scores. NSS-R change scores of the individual groups are given and its significance with the other groups in the study is indicated. Values are expressed as mean ± SEM. * P value significant <0.05. (DOCX) [file pone.0112019.s008.docx]

**Table S2**: NSS-R change scores.

| **Group** | **Comparison Group** | **P Value** |
| --- | --- | --- |
| Naïve (-.720 ± .328) | Sham | 0.372 |
|  | IS1 | 0.399 |
|  | IS3 | 0.003* |
|  | IS2 | 0.000* |
|  | IS4 | 0.000* |
| Sham (-.308 ± .322) | Naive | 0.372 |
|  | IS1 | 0.860 |
|  | IS3 | 0.025* |
|  | IS2 | 0.004* |
|  | IS4 | 0.001* |
| IS1 (-.200 ± .519) | Naive | 0.399 |
|  | Sham | 0.860 |
|  | IS3 | 0.119 |
|  | IS2 | 0.023* |
|  | IS4 | 0.004* |
| IS3 (.800 ± .367) | Naive | 0.003* |
|  | Sham | 0.025* |
|  | IS1 | 0.119 |
|  | IS2 | 0.290 |
|  | IS4 | 0.048* |
| IS2 (1.46 ± .494) | Naive | 0.000* |
|  | Sham | 0.004* |
|  | IS1 | 0.023* |
|  | IS3 | 0.290 |
|  | IS4 | 0.294 |
| IS4 (2.33 ± .670) | Naive | 0.000* |
|  | Sham | 0.001* |
|  | IS1 | 0.004* |
|  | IS3 | 0.048* |
|  | IS2 | 0.294 |

NSS-R change scores of the individual groups are given and its significance with the other groups in the study is indicated. Values are expressed as mean ± SEM. * P value significant < 0.05.
