# Supplementary material for: Identification of Serum MicroRNA Signatures for Diagnosis of Mild Traumatic Brain Injury in a Closed Head Injury Model
Source: PLoS One. 2014 Nov 7;9(11):e112019. doi: 10.1371/journal.pone.0112019 (PMC4224512; doi:10.1371/journal.pone.0112019)
Supplement: Table S3 — The horizontal activity in OF. The horizontal activity (number of beam breaks) of the individual groups is given and its significance with the other groups in the study is indicated. Values are expressed as mean ± SEM. * P value significant <0.05. (DOCX) [file pone.0112019.s009.docx]

**Table S3**: The horizontal activity in OFL.

| **Group** | **Comparison Group** | **Significance level** |
| --- | --- | --- |
| Naïve (10379.72 ± 499.03) | Sham | 0.770 |
|  | IS1 | 0.070 |
|  | IS3 | 0.001* |
|  | IS2 | 0.439 |
|  | IS4 | 0.001* |
| Sham (10564.714 ± 385.01) | Naive | 0.770 |
|  | IS1 | 0.080 |
|  | IS3 | 0.000* |
|  | IS2 | 0.237 |
|  | IS4 | 0.001* |
| IS1 (11595.69 ± 441.08) | Naive | 0.070 |
|  | Sham | 0.080 |
|  | IS3 | 0.000* |
|  | IS2 | 0.007* |
|  | IS4 | 0.000* |
| IS3 (7762.35 ± 557.93) | Naive | 0.001* |
|  | Sham | 0.000* |
|  | IS1 | 0.000* |
|  | IS2 | 0.004* |
|  | IS4 | 0.360 |
| IS2 (9855.80 ± 455.55) | Naive | 0.439 |
|  | Sham | 0.237 |
|  | IS1 | 0.007* |
|  | IS3 | 0.004* |
|  | IS4 | 0.005* |
| IS4 (6695.83 ± 1018.63) | Naive | 0.001* |
|  | Sham | 0.001* |
|  | IS1 | 0.000* |
|  | IS3 | 0.360 |
|  | IS2 | 0.005* |

The horizontal activity (number of beam breaks) of the individual groups is given and its significance with the other groups in the study is indicated. Values are expressed as mean ± SEM. * P value significant < 0.05.
