# Supplementary material for: Identification of Serum MicroRNA Signatures for Diagnosis of Mild Traumatic Brain Injury in a Closed Head Injury Model
Source: PLoS One. 2014 Nov 7;9(11):e112019. doi: 10.1371/journal.pone.0112019 (PMC4224512; doi:10.1371/journal.pone.0112019)
Supplement: Table S5 — The center time of the animals in OFL. The time spent in the center (in seconds) is given for each of the groups and its comparison with the other groups is given. Values are presented as mean ± SEM. * P value significant <0.05. (DOCX) [file pone.0112019.s011.docx]

**Table S5**: The center time of the animals in OFL.

| **Group** | **Comparison Group** | **Significance level** |
| --- | --- | --- |
| Naïve (1482.83 ± 79.15) | Sham | 0.770 |
|  | IS1 | 0.070 |
|  | IS3 | 0.001* |
|  | IS2 | 0.439 |
|  | IS4 | 0.001* |
| Sham (1385.04 ± 61.03) | Naive | 0.770 |
|  | IS1 | 0.080 |
|  | IS3 | 0.000* |
|  | IS2 | 0.237 |
|  | IS4 | 0.001* |
| IS1 (1335.22 ± 69.96) | Naive | 0.070 |
|  | Sham | 0.080 |
|  | IS3 | 0.000* |
|  | IS2 | 0.007* |
|  | IS4 | 0.000* |
| IS3 (1094.26 ± 88.60) | Naive | 0.001* |
|  | Sham | 0.000* |
|  | IS1 | 0.000* |
|  | IS2 | 0.004* |
|  | IS4 | 0.360 |
| IS2 (1450.50 ± 72.28) | Naive | 0.439 |
|  | Sham | 0.237 |
|  | IS1 | 0.007* |
|  | IS3 | 0.004* |
|  | IS4 | 0.005* |
| IS4 (1156.08 ± 160.92) | Naive | 0.001* |
|  | Sham | 0.001* |
|  | IS1 | 0.000* |
|  | IS3 | 0.360 |
|  | IS2 | 0.005* |

The time spent in the center (in seconds) is given for each of the groups and its comparison with the other groups is given. Values are presented as mean ± SEM. * P value significant < 0.05.
