# Supplementary material for: Identification of Serum MicroRNA Signatures for Diagnosis of Mild Traumatic Brain Injury in a Closed Head Injury Model
Source: PLoS One. 2014 Nov 7;9(11):e112019. doi: 10.1371/journal.pone.0112019 (PMC4224512; doi:10.1371/journal.pone.0112019)
Supplement: Table S6 — The vertical activity over the time period of the study. The vertical activity of the animals (number of beam breaks) from all the groups over the period of the study is given. Values are presented as mean ± SEM. * * P value significant <0.05. (DOCX) [file pone.0112019.s012.docx]

**Table S6**: The vertical activity over the time period of the study.

| **Time** | **Comparison Group** | **Significance level** |
| --- | --- | --- |
| Day 1 (718.20 ± 34.65) | Day 14 | 0.000 |
|  | Day 30 | 0.000 |
| Day 14 (1175.47 ± 44.15) | Day 1 | 0.000 |
|  | Day 30 | 0.623 |
| Day 30 (1157.07 ± 38.32) | Day 1 | 0.000 |
|  | Day 14 | 0.623 |

The vertical activity of the animals (number of beam breaks) from all the groups over the period of the study is given. Values are presented as mean ± SEM. * * P value significant < 0.05.
