# Supplementary material for: Identification of Serum MicroRNA Signatures for Diagnosis of Mild Traumatic Brain Injury in a Closed Head Injury Model
Source: PLoS One. 2014 Nov 7;9(11):e112019. doi: 10.1371/journal.pone.0112019 (PMC4224512; doi:10.1371/journal.pone.0112019)
Supplement: Table S7 — The vertical activity in OFL. The vertical activity of the animals (number of beam breaks) in each group and its comparison with the other groups for day 1 is given. Values are presented as mean ± SEM. * P value significant <0.05. (DOCX) [file pone.0112019.s013.docx]

**Table S7**: The vertical activity in OFL.

| **Group** | **Comparison Group** | **Significance level** |
| --- | --- | --- |
| Naïve (871.77 ± 74.96) | Sham | 0.933 |
|  | IS1 | 0.262 |
|  | IS3 | 0.000* |
|  | IS2 | 0.830 |
|  | IS4 | 0.002* |
| Sham (863.79 ± 57.79) | Naive | 0.933 |
|  | IS1 | 0.172 |
|  | IS3 | 0.000* |
|  | IS2 | 0.877 |
|  | IS4 | 0.001* |
| IS1 (984.69 ± 66.38) | Naive | 0.262 |
|  | Sham | 0.172 |
|  | IS3 | 0.000* |
|  | IS2 | 0.159 |
|  | IS4 | 0.000* |
| IS3 (451.87 ± 83.77) | Naive | 0.000* |
|  | Sham | 0.000* |
|  | IS1 | 0.000* |
|  | IS2 | 0.000* |
|  | IS4 | 0.484 |
| IS2 (849.94 ± 68.47) | Naive | 0.830 |
|  | Sham | 0.877 |
|  | IS1 | 0.159 |
|  | IS3 | 0.000* |
|  | IS4 | 0.002* |
| IS4 (329.18 ± 153.77) | Naive | 0.002* |
|  | Sham | 0.001* |
|  | IS1 | 0.000* |
|  | IS3 | 0.484 |
|  | IS2 | 0.002* |

The vertical activity of the animals (number of beam breaks) in each group and its comparison with the other groups for day 1 is given. Values are presented as mean ± SEM. * P value significant < 0.05.
