# Supplementary material for: Identification of Serum MicroRNA Signatures for Diagnosis of Mild Traumatic Brain Injury in a Closed Head Injury Model
Source: PLoS One. 2014 Nov 7;9(11):e112019. doi: 10.1371/journal.pone.0112019 (PMC4224512; doi:10.1371/journal.pone.0112019)
Supplement: Table S8 — Day 1 ASR. ASR response of the animals in each groups and its comparison with the other groups is given. Values are presented as mean ± SEM. * P value significant <0.05. (DOCX) [file pone.0112019.s014.docx]

**Table S8**: Day 1 ASR.

| **Group** | **Comparison Group** | **Significance level** |
| --- | --- | --- |
| Naïve (362.43 ± 38.31) | Sham | 0.338 |
|  | IS1 | 0.650 |
|  | IS3 | 0.019* |
|  | IS2 | 0.046* |
|  | IS4 | 0.008* |
| Sham (314.76 ± 31.50) | Naive | 0.338 |
|  | IS1 | 0.141 |
|  | IS3 | 0.096 |
|  | IS2 | 0.213 |
|  | IS4 | 0.028* |
| IS1 (386.65 ± 36.91) | Naive | 0.650 |
|  | Sham | 0.141 |
|  | IS3 | 0.005* |
|  | IS2 | 0.014* |
|  | IS4 | 0.003* |
| IS3 (225.39 ± 43.18) | Naive | 0.019* |
|  | Sham | 0.096 |
|  | IS1 | 0.005* |
|  | IS2 | 0.665 |
|  | IS4 | 0.276 |
| IS2 (251.16 ± 39.72) | Naive | 0.046* |
|  | Sham | 0.213 |
|  | IS1 | 0.014* |
|  | IS3 | 0.665 |
|  | IS4 | 0.158 |
| IS4 (127.01 ± 78.41) | Naive | 0.008* |
|  | Sham | 0.028* |
|  | IS1 | 0.003* |
|  | IS3 | 0.276 |
|  | IS2 | 0.158 |

ASR response of the animals in each groups and its comparison with the other groups is given. Values are presented as mean ± SEM. * P value significant <0.05.
