# Supplementary material for: Identification of Serum MicroRNA Signatures for Diagnosis of Mild Traumatic Brain Injury in a Closed Head Injury Model
Source: PLoS One. 2014 Nov 7;9(11):e112019. doi: 10.1371/journal.pone.0112019 (PMC4224512; doi:10.1371/journal.pone.0112019)
Supplement: Table S9 — Day 14 ASR. ASR response of the animals in each groups and its comparison with the other groups is given. Values are presented as mean ± SEM. * P value significant <0.05. (DOCX) [file pone.0112019.s015.docx]

**Table S9**: Day 14 ASR.

| **Group** | **Comparison Group** | **Significance level** |
| --- | --- | --- |
| Naïve (333.72 ± 36.72) | Sham | 0.365 |
|  | IS1 | 0.114 |
|  | IS3 | 0.998 |
|  | IS2 | 0.408 |
|  | IS4 | 0.009* |
| Sham (290.52 ± 30.20) | Naive | 0.365 |
|  | IS1 | 0.415 |
|  | IS3 | 0.401 |
|  | IS2 | 0.988 |
|  | IS4 | 0.001* |
| IS1 (252.51 ± 35.38) | Naive | 0.114 |
|  | Sham | 0.415 |
|  | IS3 | 0.138 |
|  | IS2 | 0.477 |
|  | IS4 | 0.000* |
| IS3 (333.59 ± 41.39) | Naive | 0.998 |
|  | Sham | 0.401 |
|  | IS1 | 0.138 |
|  | IS2 | 0.442 |
|  | IS4 | 0.011* |
| IS2 (289.78 ± 38.08) | Naive | 0.408 |
|  | Sham | 0.988 |
|  | IS1 | 0.477 |
|  | IS3 | 0.442 |
|  | IS4 | 0.002* |
| IS4 (554.82 ± 75.15) | Naive | 0.009* |
|  | Sham | 0.001* |
|  | IS1 | 0.000* |
|  | IS3 | 0.011* |
|  | IS2 | 0.002* |

ASR response of the animals in each groups and its comparison with the other groups is given. Values are presented as mean ± SEM. * P value significant <0.05.
