# Supplementary material for: Identification of Serum MicroRNA Signatures for Diagnosis of Mild Traumatic Brain Injury in a Closed Head Injury Model
Source: PLoS One. 2014 Nov 7;9(11):e112019. doi: 10.1371/journal.pone.0112019 (PMC4224512; doi:10.1371/journal.pone.0112019)
Supplement: Table S10 — Average Ct values for the common miRNAs. Average Ct value for each of the miRNA in the injury and the sham control group indicate their abundance in the sample. High expression = Ct ranging from 11–15; Good expression = Ct ranging from 16–20; Moderate expression = Ct ranging from 21–25; Low Expression = Ct ranging from 26–30; and Rare expression = Ct ranging from 31–35. (DOCX) [file pone.0112019.s016.docx]

**Table S10:** Average Ct values for the common miRNAs.

| **MiRNA** | **IS1** | **SD** | **IS2** | **SD** | **IS3** | **SD** | **IS4** | **SD** | **Sham** | **SD** |
| --- | --- | --- | --- | --- | --- | --- | --- | --- | --- | --- |
| mmu-miR-574-3p | 18.62 | 2.13 | 18.37 | 1.11 | 16.94 | 0.61 | 17.42 | 1.12 | 20.37 | 0.49 |
| hsa-miR-214 | 19.41 | 1.92 | 18.76 | 1.03 | 17.97 | 1.02 | 17.70 | 1.05 | 21.40 | 0.86 |
| mmu-miR-106b | 19.84 | 2.45 | 19.61 | 1.42 | 20.05 | 1.11 | 19.43 | 1.25 | 18.37 | 0.93 |
| mmu-miR-214 | 21.48 | 2.57 | 20.99 | 1.40 | 19.36 | 0.60 | 19.50 | 1.09 | 23.35 | 0.55 |
| mmu-miR-199a-3p | 22.92 | 2.25 | 22.40 | 1.12 | 21.43 | 0.91 | 20.78 | 1.46 | 24.04 | 0.69 |
| mmu-miR-218 | 23.39 | 2.58 | 22.28 | 1.41 | 21.78 | 1.33 | 21.80 | 1.43 | 24.65 | 0.97 |
| mmu-miR-434-3p | 23.73 | 2.00 | 22.89 | 1.73 | 21.94 | 0.76 | 21.73 | 1.74 | 25.15 | 0.86 |
| hsa-miR-106b# | 24.28 | 2.22 | 24.05 | 1.47 | 24.07 | 1.12 | 23.02 | 0.96 | 23.03 | 0.78 |
| mmu-miR-337-5p | 25.36 | 2.63 | 25.28 | 1.65 | 23.51 | 0.92 | 24.21 | 1.28 | 27.14 | 0.65 |
| mmu-miR-31 | 25.49 | 1.50 | 25.92 | 1.40 | 24.55 | 1.14 | 24.39 | 1.19 | 22.75 | 3.13 |
| mmu-miR-671-3p | 26.91 | 2.39 | 26.81 | 0.93 | 24.82 | 1.00 | 24.81 | 1.37 | 28.29 | 0.61 |
| mmu-miR-376a | 30.20 | 1.06 | 30.97 | 1.12 | 30.08 | 0.94 | 30.11 | 2.93 | 36.88 | 3.45 |
| rno-miR-196c | 32.20 | 6.15 | 31.08 | 6.97 | 29.81 | 5.06 | 31.04 | 7.17 | 26.26 | 0.95 |

Average Ct value for each of the miRNA in the injury and the sham control group indicate their abundance in the sample. High expression= Ct ranging from 11-15; Good expression = Ct ranging from 16-20; Moderate expression = Ct ranging from 21-25; Low Expression = Ct ranging from 26-30; and Rare expression = Ct ranging from 31-35.
