# Supplementary material for: Identification of Serum MicroRNA Signatures for Diagnosis of Mild Traumatic Brain Injury in a Closed Head Injury Model
Source: PLoS One. 2014 Nov 7;9(11):e112019. doi: 10.1371/journal.pone.0112019 (PMC4224512; doi:10.1371/journal.pone.0112019)
Supplement: Table S11 — MiRNAs present only in the injury groups that demonstrate behavior changes. Nineteen miRNAs were significantly modulated in the injury group that showed significant behavior alterations. Of these 14 were up regulated and 5 were down regulated. Two miRNAs, mmu-miR-487b and mmu-miR-218-1* were expressed only in the injured animals and not in the sham-injured animals i.e., “calibrator not detected”. For all other miRNAs Ct value was <36 in both, the injured and the sham-injured groups. Values are given as log10 of the fold change (2−(mean ΔΔCt)). (DOCX) [file pone.0112019.s017.docx]

**Table S11:** MiRNAs present only in the injury groups that demonstrate behavior changes.

| **S.No.** | **MiRNA** | **IS2** | **P value** | **IS3** | **P value** | **IS4** | **P value** |
| --- | --- | --- | --- | --- | --- | --- | --- |
| 1 | mmu-miR-487b | 1.906 | 0.002 | 2.013 | 0.001 | 1.865 | 0.003 |
| 2 | mmu-miR-218-1* | 1.049 | 0.034 | 1.105 | 0.030 | 0.982 | 0.049 |
| 3 | mmu-miR-384-5p | 0.731 | 0.007 | 0.777 | 0.001 | 0.737 | 0.026 |
| 4 | mmu-miR-667 | 0.693 | 0.005 | 0.908 | 0.001 | 1.194 | 0.001 |
| 5 | mmu-miR-122 | 0.608 | 0.002 | 1.240 | 0.000 | 1.408 | 0.000 |
| 6 | mmu-miR-34b-3p | 0.598 | 0.003 | 0.537 | 0.000 | 0.914 | 0.001 |
| 7 | mmu-miR-872* | 0.556 | 0.009 | 0.603 | 0.003 | 0.584 | 0.009 |
| 8 | mmu-miR-485-3p | 0.534 | 0.026 | 0.835 | 0.001 | 0.842 | 0.000 |
| 9 | mmu-miR-204 | 0.442 | 0.014 | 0.324 | 0.004 | 0.769 | 0.000 |
| 10 | mmu-miR-132 | 0.396 | 0.043 | 0.480 | 0.006 | 0.611 | 0.007 |
| 11 | mmu-miR-152 | 0.361 | 0.016 | 0.439 | 0.002 | 0.399 | 0.010 |
| 12 | mmu-miR-212 | 0.315 | 0.039 | 0.289 | 0.024 | 0.410 | 0.032 |
| 13 | mmu-miR-192 | 0.299 | 0.002 | 0.644 | 0.004 | 0.574 | 0.000 |
| 14 | mmu-miR-145 | 0.247 | 0.028 | 0.532 | 0.001 | 0.555 | 0.001 |
| 15 | mmu-miR-19b | -0.252 | 0.036 | -0.292 | 0.013 | -0.268 | 0.022 |
| 16 | mmu-miR-18a | -0.335 | 0.022 | -0.434 | 0.002 | -0.319 | 0.030 |
| 17 | mmu-miR-451 | -0.418 | 0.015 | -0.777 | 0.000 | -0.513 | 0.010 |
| 18 | mmu-miR-26b | -0.529 | 0.032 | -0.635 | 0.007 | -0.574 | 0.015 |
| 19 | hsa-miR-875-5p | -3.021 | 0.041 | -3.161 | 0.025 | -3.749 | 0.010 |

Nineteen miRNAs were significantly modulated in the injury group that showed significant behavior alterations. Of these 14 were up regulated and 5 were down regulated. Two miRNAs, mmu-miR-487b and mmu-miR-218-1* were expressed only in the injured animals and not in the sham-injured animals *i.e.,* “calibrator not detected”. For all other miRNAs Ct value was <36 in both, the injured and the sham-injured groups. Values are given as log10 of the fold change (2^-(mean ∆∆Ct)^).
