# Supplementary material for: Identification of Serum MicroRNA Signatures for Diagnosis of Mild Traumatic Brain Injury in a Closed Head Injury Model
Source: PLoS One. 2014 Nov 7;9(11):e112019. doi: 10.1371/journal.pone.0112019 (PMC4224512; doi:10.1371/journal.pone.0112019)
Supplement: Table S12 — MiRNA present only in the injury groups that do not demonstrate behavior changes. Two miRNAs, miR-297a* and miR-181c were found to be significantly modulated in the IS1 injury group, which did not demonstrate any significant neurobehavioral and cognitive changes as compared to the sham injury group. “Calibrator not detected” is the miRNA which is expressed only in the injured animals and not in the un-injured sham animals. “Target not detected” is the miRNA that was expressed only in the un-injured sham animals and not in the injured animals. In such case the Ct value for non-detected miRNA was taken as 40 followed by the fold change and statistical analysis. (DOCX) [file pone.0112019.s018.docx]

**Table S12**: MiRNA present only in the injury groups that do not demonstrate behavior changes.

| **MiRNA** | **P value** | **Ct Status** | **Fold Change (Log10)** |
| --- | --- | --- | --- |
| mmu-miR-297a* | 0.03 | Calibrator not detected | 1.55 |
| mmu-miR-181c | 0.02 | Target not detected | -1.65 |

Two miRNAs, miR-297a* and miR-181c were found to be significantly modulated in the IS1 injury group, which did not demonstrate any significant neurobehavioral and cognitive changes as compared to the sham injury group. “Calibrator not detected” is the miRNA which is expressed only in the injured animals and not in the un-injured sham animals. “Target not detected” is the miRNA that was expressed only in the un-injured sham animals and not in the injured animals. In such case the Ct value for non-detected miRNA was taken as 40 followed by the fold change and statistical analysis.
