# Supplementary material for: Identification of Serum MicroRNA Signatures for Diagnosis of Mild Traumatic Brain Injury in a Closed Head Injury Model
Source: PLoS One. 2014 Nov 7;9(11):e112019. doi: 10.1371/journal.pone.0112019 (PMC4224512; doi:10.1371/journal.pone.0112019)
Supplement: Table S13 — Selection of the endogenous control miRNA. The most stable miRNA were selected based on their Ct values across the samples (n = 30). Ct for each miRNA from all the samples, irrespective of the injury or control group, were taken and Ct values with a SD <1 were selected. Mean Ct were calculated to determine the abundance of miRNAs in the serum samples. To ensure that there are no outliers, median Ct was also calculated. MiRNA that showed similar mean and median Ct values were then selected. MiRNA that show more abundance i.e., Ct <25 were considered for determining a candidate stable endogenous miRNA in validation experiments. (DOCX) [file pone.0112019.s019.docx]

**Table S13**: Selection of the endogenous control miRNA.

| **S. No.** | **Detector** | **Mean Ct** | **Median Ct** | **SD** |
| --- | --- | --- | --- | --- |
| 1 | mmu-miR-1937b | 14.91 | 14.81 | 0.90 |
| 2 | mmu-miR-486 | 15.39 | 15.02 | 0.92 |
| 3 | mmu-miR-16 | 15.54 | 15.52 | 0.91 |
| 4 | mmu-miR-1937c | 15.79 | 15.95 | 0.78 |
| 5 | mmu-miR-1274a | 15.93 | 15.64 | 0.93 |
| 6 | mmu-miR-106a | 17.93 | 17.94 | 0.85 |
| 7 | mmu-miR-17 | 17.94 | 17.90 | 0.84 |
| 8 | mmu-miR-93 | 19.73 | 19.70 | 0.89 |
| 9 | mmu-miR-2146 | 20.44 | 20.38 | 0.99 |
| 10 | hsa-miR-93# | 21.03 | 20.95 | 0.87 |
| 11 | mmu-miR-20b | 22.59 | 22.58 | 0.94 |
| 12 | hsa-miR-421 | 23.34 | 23.00 | 0.79 |
| 13 | mmu-miR-18a# | 25.04 | 24.82 | 0.81 |
| 14 | mmu-miR-2182 | 25.64 | 25.64 | 0.97 |
| 15 | mmu-miR-1930 | 25.92 | 25.84 | 0.81 |
| 16 | rno-miR-148b-5p | 27.19 | 26.98 | 1.00 |
| 17 | mmu-miR-29b# | 27.36 | 27.27 | 0.62 |
| 18 | mmu-miR-467c | 27.61 | 27.36 | 0.95 |

The most stable miRNA were selected based on their Ct values across the samples (n=30). Ct for each miRNA from all the samples, irrespective of the injury or control group, were taken and Ct values with a SD < 1 were selected. Mean Ct were calculated to determine the abundance of miRNAs in the serum samples. To ensure that there are no outliers, median Ct was also calculated. MiRNA that showed similar mean and median Ct values were then selected. MiRNA that show more abundance *i.e.,* Ct<25 were considered for determining a candidate stable endogenous miRNA in validation experiments.
